# π-Extended perylene diimide double-heterohelicenes as ambipolar organic semiconductors for broadband circularly polarized light detection

**DOI:** 10.1038/s41467-020-20390-y

**Published:** 2021-01-08

**Authors:** Li Zhang, Inho Song, Jaeyong Ahn, Myeonggeun Han, Mathieu Linares, Mathieu Surin, Hui-Jun Zhang, Joon Hak Oh, Jianbin Lin

**Affiliations:** 1 Department of Chemistry, College of Chemistry and Chemical Engineering, MOE Key Laboratory of Spectrochemical Analysis and Instrumentation, Xiamen University, Xiamen, 361005 China; 2 School of Chemical and Biological Engineering, Institute of Chemical Processes, Seoul National University, 1 Gwanak-ro, Gwanak-gu, Seoul 08826 Korea; 3 Department of Chemical Engineering, Pohang University of Science and Technology (POSTECH), Gyeongbuk, Pohang 37673 Korea; 4 Laboratory of Organic Electronics and Scientific Visualization Group, ITN, Campus Norrköping; Swedish e-Science Research Centre (SeRC), Linköping University, Linköping, SE-581 83 Sweden; 5 Laboratory for Chemistry of Novel Materials, Centre of Innovation and Research in Materials and Polymers (CIRMAP), University of Mons – UMONS, 20 Place du Parc, Mons, B-7000 Belgium

**Keywords:** Self-assembly, Nonlinear optics, Self-assembly

## Abstract

Despite great challenges, the development of new molecular structures with multiple and even conflicting characteristics are eagerly pursued for exploring advanced applications. To develop high-performance chiral organic semiconducting molecules, a distorted π-system is required for strong coupling with circularly polarized light (CPL), whereas planar π-stacking systems are necessary for high charge-carrier mobility. To address this dilemma, in this work, we introduce a skeleton merging approach through distortion of a perylene diimide (PDI) core with four fused heteroaromatics to form an *ortho*-π-extended PDI double-[7]heterohelicene. PDI double helicene inherits a high dissymmetry factor from the helicene skeleton, and the extended π-planar system concurrently maintains a high level of charge transport properties. In addition, *ortho*-π-extension of the PDI skeleton brings about near-infrared (NIR) light absorption and ambipolar charge transport abilities, endowing the corresponding organic phototransistors with high photoresponsivity of 450 and 120 mA W^−1^ in *p*- and *n*-type modes respectively, along with a high external quantum efficiency (89%) under NIR light irradiations. Remarkably, these multiple characteristics enable high-performance broadband CPL detections up to NIR spectral region with chiral organic semiconductors.

## Abstract (web summary)

In organic semiconducting molecules materials, distorted π-systems enable strong coupling with circular polarized light while planar π-stacking systems are necessary for high charge-carrier mobility. Here, the authors address this dilemma by introducing a skeleton merging approach through distortion of a perylene diimide core with four fused heteroaromatics to form a π-extended double helicene.

## Introduction

The bright future of organic semiconducting materials hinges upon the rational design at the molecular and supramolecular levels, which can be directly reflected by their diverse optoelectronic properties and morphologies^1,2^. A driving role in this setting falls on multiple and sometimes conflicting requirements that have to be fulfilled simultaneously on molecular designs. In recent years, distinctive chiroptical properties arising from interactions of chiral chromophores with electromagnetic fields have led to a growing recognition that active chiral components would provide additional functions in optoelectronic devices^3,4^. However, the search for chiral organic semiconductors is still at a very early stage, because principles for design of chiral chromophores with highly-efficient absorption of circularly polarized light (CPL) have been in conflict with the requirements for improved charge transport properties.

As a typical example of chiral π-systems, helicenes have a large chiral pitch matching a specific handedness of CPL, which can largely enhance the light–matter interactions^5^. As a result, helicenes exhibits excellent chiroptical performances, such as high circularly polarized absorption and luminescence values^6–16^. Unfortunately, the exploration of their electronic properties has been held back on account of the weak electron delocalization in small π-planes and the loose molecular packing in solid state caused by the strongly distorted screw-shaped aromatic rings. In 2013, Campbell and co-workers^17^ reported the first CPL detection by a chiral organic semiconductor phototransistor employing chiral helicene as an active layer. A specific photoresponse to CPL was achieved albeit at the expense of rather low charge-carrier mobility (~10^−4^ cm^2^ V^−1^ s^−1^) and photoresponsivity (quantum efficiency ~ 0.1%). Notably, due to the disturbed π-conjugation, helicenes generally show chiroptical properties in only UV and near blue part of the visible light spectrum (<400 nm), which prevents advanced applications based on near-infrared (NIR) light absorption. In contrast, as an important class of organic dyes, perylene diimides (PDIs) have received great attention for applications as organic optoelectronics due to their unique features such as high charge-carrier mobilities, favorable electron affinities, air stability, and facile molecular design^18–20^. Recently, even several PDI-based helical ribbons^21,22^ and other twisted structures^23,24^ with high charge-carrier mobilities were reported. In 2017, we employed a supramolecular assembly of PDIs possessing chiral side groups for chiroptical sensing^25^. This system exhibited a broad UV–vis absorption range (<~600 nm) and high photoresponsivity, but very poor CPL selectivity owing to the chirality in the side chains that provides only weak perturbations to the electronic transitions of the main chromophores.

**Fig. 1:**
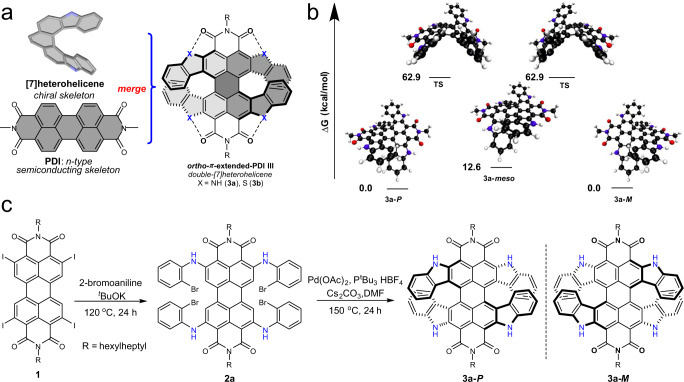
Molecule design and synthesis. **a** Schematic representation of the skeleton merging approach. **b** Potential energy surface of the isomerization process between **3a-*P***/***M*** and **3a-*meso*** conformations, calculated by DFT calculations at the ωB97Xd/6–31+G(d) level (hexylheptyl groups have been replaced by methyl groups to simplify the calculations, carbon = black, hydrogen = white, nitrogen = blue, oxygen = red). **c** Synthesis of tetraindole-fused PDIs **3a**.

Herein, we introduce a skeleton merging approach to combine both helicene and PDI to afford *ortho*-π-extended PDI double-[7]heterohelicenes, which encompass the high chiroptical and electronic properties from both parent skeletons and offer new properties such as NIR light absorption and ambipolar charge-carrier transport properties (Fig. 1a). The corresponding organic phototransistor exhibits comparable external quantum efficiency (EQE) among thin-film-based organic CPL detectors reported to date^17,26,27^. Especially, its photoresponsivity is even superior to the reported chiral NIR light detectors based on silicon and chiral plasmonic-patterned electrodes^28^. The *ortho*-π-extended PDI double-heterohelicene allows CPL detection using chiral organic semiconductors at NIR spectral region for the first time, together with excellent thermal stability. This broadband CPL detection could provide unprecedented opportunities in optoelectronics, by introducing a new functionality to various prospective applications, including motion detection, remote sensing, health monitoring, photoplethysmogram sensor, spectrometric analysis, and night vision.

## Results

### Design and synthesis

Twisting of the PDI core engendered by steric repulsion between the encumbered *bay*-substituents leads to the occurrence of atrop-enantiomers (*P-* and *M-*enantiomers). However, despite significant distortion in these *bay*-substituted^29^ and *ortho*-fused^30,31^ PDI systems, a fast interconversion process between the *P-* and *M-*enantiomers prevents the isolation of enantiopure derivatives for further applications. As the activation parameters for conformational chiral systems are influenced by the size of the substituents, we speculated that annulation of four indole/benzothiophene rings to perylene skeleton with heteroatom at *ortho*-position should lead to conformationally stable double-[7]heterohelicene **3a** (X = NH) and **3b** (X = S) with overlapping terminal aromatic rings (Fig. 1a). The rigidity of the π-skeleton can be further enhanced via the hydrogen bonds^32^ and chalcogen bonds^33^. The incorporation of heteroatoms in helicenes would offer the possibility to tune their band gap. Additionally, the donor (heteroaromatics)–acceptor (PDI) fused architecture might show ambipolar charge transport properties, which would improve the performance of electronic circuits.

We began our studies by evaluating the isomerization process from *P-*/*M-*enantiomers to the *meso-*conformer through a proposed transition state by DFT calculations (using 6–31 + G(d) basis set with the ωB97Xd^34^ functional to include dispersion corrections). The *P-*/*M-*enantiomers of **3a**, **3b** bearing methyl substituents at imide positions are more stable than their *meso-*forms by 12.6 and 13.1 kcal mol^−1^, respectively (Fig. 1b and Supplementary Fig. 1). Accordingly, the isomerization barriers were estimated to be 62.9 and 65.2 kcal mol^−1^, respectively, which are notably high enough for further separation by chiral high-performance liquid chromatography (HPLC).

Encouraged by this result, we have synthesized **3a** on the basis of *ortho*-mono-indole fused PDI **3a′** with a twist angle of 24°, which were prepared using a mild and regioselective Rh(III)-catalyzed method (Supplementary Fig. 2)^35^. Before this work, we have noticed that two related compounds have already been synthesized by Wang and co-workers^36^ with tetrabromotetrachloro-perylene-3,4:9,10-tetracarboxylic acid dianhydride (Br_4_Cl_4_-PTCDA). However, the harsh reaction conditions and tedious synthetic procedures involving Br_4_Cl_4_-PTCDA constrained further explorations of their chiral properties. As depicted in Fig. 1c, in our case, through nucleophilic aromatic substitution reactions of **1**, intermediate **2a** was produced in 81% yield. After optimizing palladium-catalyzed intramolecular C–H activation conditions, tetraindole-fused PDI **3a** could be obtained with a 51% yield when **2a** was treated with Pd(OAc)_2_ catalyst. Due to its twisted structure, **3a** displays enhanced solubility in common organic solvents, such as dichloromethane (DCM), chloroform, and tetrahydrofuran. In NMR measurements, the aromatic protons assigned to the indole of **3a** are significantly shifted upfield compared to **3a′**, reflecting the π-overlap at the terminal rings of **3a**.

### Photophysical property characterization

**Fig. 2:**
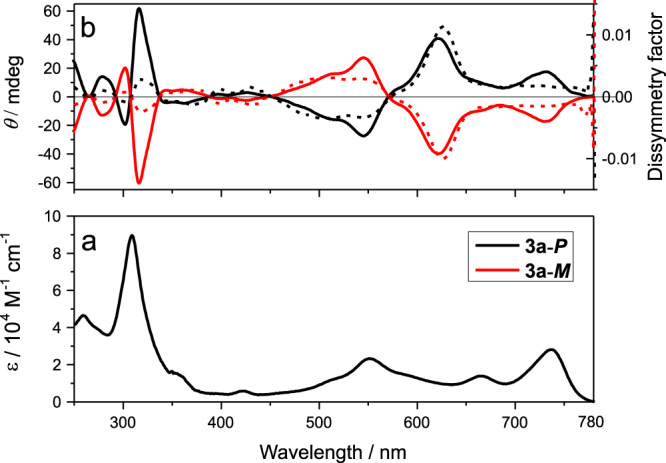
Photophysical property characterization. **a** Absorption and **b** CD spectrum/dissymmetry factor (*g*_abs_) of **3a** (10 μM in chloroform, solid line: CD, dashed line: *g*_abs_).

By using chiral HPLC, the two enantiomers of **3a** could be completely separated (47% **3a**-***P***, 50% **3a**-***M***). Their optophysical properties were studied by absorption and CD spectroscopy (Fig. 2). Compared to **1**, the substantial red shifts (~100 nm, Supplementary Fig. 4) of **3a′** indicate an effective expansion of the π systems even with one fused heteroaromatic ring. The introduction of four fused rings induced a further bathochromic shift to NIR with a maximum of **3a** at 737 nm (Fig. 2a). The enantiomers were subsequently characterized by CD spectroscopy, which displays a perfect mirror image of the enantiomers (Fig. 2b). It is noteworthy that the CD signal reaches the NIR region (~780 nm). High dissymmetry in the PDI double helicene is as expected, and the ***g***_**abs**_ factor of **3a** (628 nm: 0.014) is higher than that for a classical hexahelicene (0.009)^16^. More importantly, **3a** still shows high ***g***_**abs**_ factor even above a wavelength of 700 nm, which enables its application in advanced chiroptical devices. Excitonic couplet at about 309 nm for **3a** can be attributed to the excitonic interaction of the two twisted π-extended naphthalene imide units of the PDI core. For a more reliable configurational assignment of the enantiomers, TD-DFT calculations were performed to calculate the CD spectra. The signs of the calculated CD bands clearly point to the assignment of (+/−) signals at low energy for **3a-*P*** enantiomer, and (−/+) signals for **3a-*M*** enantiomer (Supplementary Fig. 6). Note that the first band at low energy can be attributed to the deformation of the PDI core of the molecule from a planar conformation with a twist of +33.6° and −33.6° for the *P* and *M* conformers, respectively (Supplementary Fig. 7). In addition, the diphenyl ether solution of **3a**-***P*** was heated at different temperatures and the isomerization was not observed even heated up to 523 K for 2 h (Supplementary Fig. 8), suggesting the remarkable conformational stability of **3a-*P***/***M*** as predicted by the DFT calculation. From cyclic voltammetry measurements of **3a** in DCM solutions (Supplementary Table 1), the corresponding HOMO/LUMO energy levels are estimated as −5.31/−3.75 eV. Based on the HOMO/LUMO energy levels, and the small band gap (1.56 eV), **3a** should favor balanced ambipolar charge injection and CPL detection in the NIR range (see below).

### Morphological and chiroptical study of thin films

**Fig. 3:**
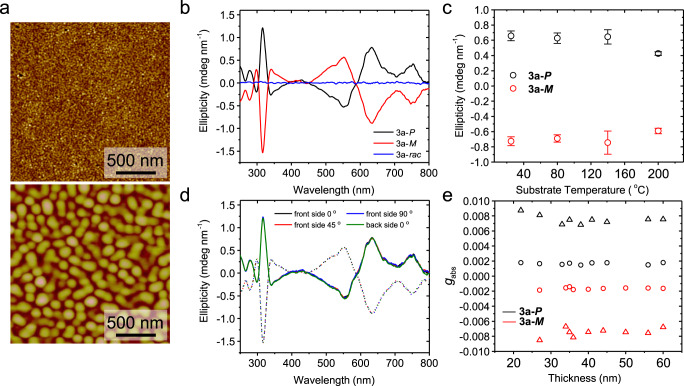
Morphological chiroptical study of thin film. **a** AFM topography images of **3a-*P*** films deposited at a substrate temperature of RT (top) and 140 °C (bottom). **b** Thickness normalized ellipticity of **3a** thin films deposited at 140 °C. **c** Thickness normalized ellipticity values at 635 nm for **3a-*P*/*M*** thin films prepared at different substrate temperature conditions. Error bars mean standard deviations. **d** Thickness normalized ellipticity of **3a-*P*/*M*** thin films deposited at 140 °C after sample flipping (back side) and sample rotation. Solid and dotted lines mean **3a-*P*** and **3a-*M***, respectively. **e** Estimated *g*_abs_ value of **3a-*P*/*M*** thin films evaporated at 140 °C. Circles and triangles mean *g*_abs_ values at 740 and 635 nm, respectively.

To elucidate molecular packing characteristics in terms of morphology in the aggregated solid films, we investigated the morphologies of **3a-*P*** thin films fabricated by a thermal evaporation process. Forty-nm-thick PDI derivatives thin films were thermally evaporated onto *n*-octadecyltrimethoxysilane (OTS)-modified SiO_2_/Si substrates under high vacuum (~10^−6^ Torr) at different substrate temperatures (*T*_s_). Scanning electron microscope (SEM) analysis on the **3a-*P*** solid films displayed dense granular crystalline domains, when the **3a-*P*** molecules were solidified at an optimized *T*_s_ of 140 °C (Supplementary Fig. 9a). However, SEM measurement could not distinguish the morphology of **3a-*P*** solid films prepared at a *T*_s_ of room temperature (RT), because of their very smooth and small domains (Supplementary Fig. 9b). To further investigate the grains at the nanoscale, atomic force microscopy (AFM) analysis was conducted. Figure 3a presents the height images (2 × 2 µm^2^ scan) of the **3a-*P*** thin films prepared at different *T*_s_. AFM analysis revealed that the optimally evaporated thin films at 140 °C consisted of dense granular grains, which are large (~100 nm) and well-connected each other (Fig. 3a, bottom), as shown in the aforementioned SEM results. However, the grain size decreased as the **3a-*P*** molecules were solidified on the substrate at RT condition (Fig. 3a, top, 80 °C in Supplementary Fig. 10b). Although they showed dense, granular morphology similar to those prepared at higher *T*_s_, the films displayed smaller domains of 10–30 nm. The much larger domains in solid films prepared at the optimal *T*_s_ originated from the efficient diffusion of chiral molecules on the substrate and a dominant aggregation process, which plays a key role in determining the electrical properties such as a field-effect mobility of organic thin-film transistors. In addition, we also observed that too high substrate temperature process (i.e., above 200 °C) causes an island-type aggregation, leading to absence of continuity in the channel and therefore very limited charge transport (Supplementary Fig. 10d).

CD spectroscopy was used to further investigate the chiroptical properties of the thin films of **3a** (Fig. 3b). Interestingly, we observed similar CD spectra to the monomer state in solution, with slightly red-shifted sharp peaks. These similar properties indicate that the thin films showed the molecular chirality rather than supramolecular chirality with the intermolecular exciton coupling in the aggregated state. Indeed, we confirmed the aggregated thin films retained the vibrational progression of monomer state from UV–vis measurement (Supplementary Fig. 11), which is a typical phenomenon found in loosely packed PDI molecules with a weak intermolecular interaction in solid state^37^.

In addition, we confirmed the temperature-dependent CD behaviors of the evaporated thin films on the substrate (Fig. 3c). Unlike the morphological trend (grain size), the ellipticity was almost constant irrespective of the substrate temperature, except the island-type thin films prepared at a very high temperature (200 °C). To study the molecular packing and crystallinity in aggregated state, the **3a** thin films were investigated using the out-of-plane X-ray diffraction (XRD; Supplementary Fig. 12). **3a** thin films did not exhibit any diffraction peaks except the peaks from SiO_2_ as substrate regardless of the chiral composition of organic molecules or evaporated conditions, leading to the absence of anisotropic orientation. AFM and XRD analyses both revealed almost no preferred orientation of **3a** molecules in thin films. Unlike strong intermolecular orientation of PDI semiconductors using π–π stacking^38–42^, **3a** thin films exhibited completely circular or spherical grains, not rod type ones, which indicates their weak intermolecular interactions in the aggregated states, related to loosely, isotropic molecular packing.

Furthermore, we tried to measure CD spectra by considering the azimuthal sample rotation and sample flipping for the investigation on the origin of the CD effect in our solid system and the precise estimation of dissymmetry *g*-factor for thin films in the conventional CD measurements. Indeed, the strong linear dichroism/birefringence and their synergistic effect can strongly affect CD results. In aggregated solid state, the preferential molecular ordering and anisotropic crystalline nature generate the cross term between the linear dichroism and birefringence, leading to significant contribution to the CD signal^43^. First, we checked that thin films of **3a** do not have any locally anisotropic and linearly birefringent domains by observing microscope images with crossed linear polarizers (Supplementary Fig. 13). We noticed that there are no birefringent domains on a black background. In addition, we observed the CD spectra of the evaporated thin films by rotating the azimuthal angle of the mounted sample around the optical axis of the incident light to remove artificial CD based on the macroscopic anisotropic effect of the sample. Figure 3d and Supplementary Fig. 14 display almost negligible difference in the CD spectra, which indicates the absence of macroscopic structural ordering or any preferential orientation in parallel to the glass surface, in line with the XRD results. In addition, we tested the CD spectra by flipping the sample by 180° with respect to the vertical axis^43^. We confirmed that there is no change of CD spectra in the entire spectral range, indicating that the chirality in the thin film originates from the intrinsic chirality of the helical molecules, and not a product of linear dichroism and birefringence^44,45^. Based on the recorded ellipticity of thin films, we estimated the *g*_abs_ value depending on the film thickness (Fig. 3e). We observed the almost identical, thickness-independent dissymmetry factors, indicative of intrinsic chiroptical properties in aggregated solid systems. They exhibited relatively lower *g*_abs_ values than those in the solution state, which is indicative of no amplification of chirality via supramolecular chirality^27,46,47^.

### Device fabrication and electrical performance

**Fig. 4:**
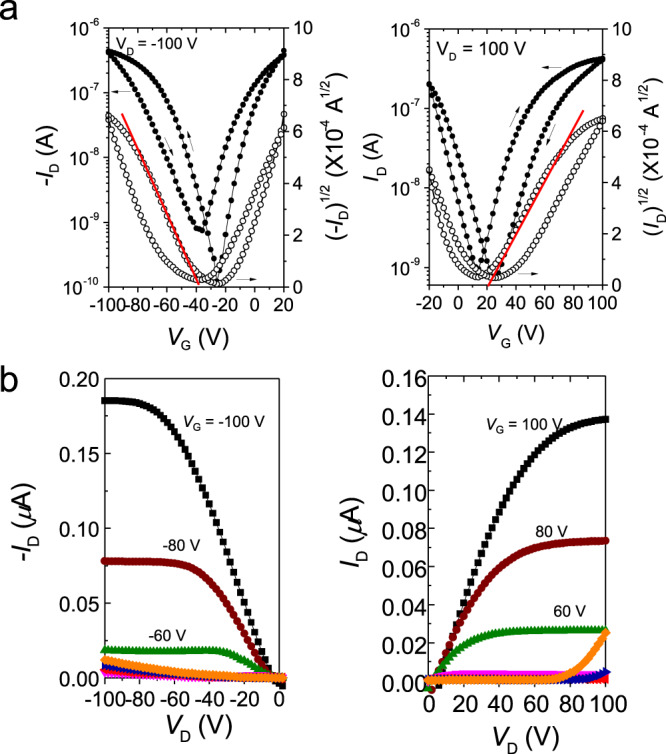
Electrical performances. **a** Transfer characteristics of OFETs based on **3a-*P*** films deposited at a *T*_s_ of 140 °C in *p*-type (left) and *n*-type (right) modes. **b** Output characteristics of OFETs based on **3a-*P*** films deposited at a *T*_s_ of 140 °C in *p*-type (left) and *n*-type (right) modes. Solid red lines indicate the region for mobility estimation.

To further investigate the electrical properties of PDI double helicenes as chiral organic semiconductors, thin-film-based organic field-effect transistors (OFETs) were fabricated and their electrical behaviors were examined. The experimental details of the OFET fabrication and measurements are given in the Supplementary Information. Due to their unstable air stability, we tested the transfer and output characteristics in the vacuum chamber. The transfer and output characteristics are shown in Fig. 4 and OFET characteristics are summarized in Supplementary Table 2.

Each enantiomer exhibited almost identical electrical properties regardless of the chirality of molecules (***P*** or ***M***). Interestingly, **3a-*P***/***M*** films prepared at an optimized *T*_s_ of 140 °C displayed ambipolar field-effect behaviors with typical V-shaped transfer curves. They exhibited high charge-carrier mobility with average hole and electron mobility of 2.1 × 10^−3^ and 1.7 × 10^−3^ cm^2^ V^−1^ s^−1^, respectively (**3b′** and **3b** were also synthesized. **3b-*P***/***M*** films at an optimized *T*_s_ of 140 °C exhibited normal *n*-type OFET characteristics with an average electron mobility of 2.5 × 10^−5^ cm^2^ V^−1^ s^−1^, for details see Supplementary Figs. 2, 3 and 15 and Table 2). The charge-carrier mobility was calculated using the slope from a plot of the square root of drain current against gate voltage in the transfer curve at the region marked by red solid line, because they showed non-linearity at high gate voltage (*V*_G_) region, which was confirmed by the characteristics of other organic TFTs^48–50^. However, they showed no kink at the low *V*_G_ region, leading to the ideal operation with bias-independent charge-carrier mobilities. In addition, they showed good bias stability and relatively small charge traps at the organic semiconductor–dielectric interface in transfer characteristics with a hysteresis test. The well-balanced hole and electron mobilities makes **3a** very attractive to be applied in complementary circuits. The comparison of chiroptical and electrical performance of **3a** with those of previously reported helical organic semiconductors is summarized in Supplementary Table 3. Remarkably, our *ortho*-π-extended PDIs substantially extends the chiroptically responsive wavelength up to the NIR region (~780 nm) and exhibits the moderate charge-carrier mobilities with well-balanced ambipolarity in thin-film-type OFETs, in comparison with previously reported helical organic semiconductors^17,31,51–56^. In addition to **3a-*P***/***M***, **3a-*rac*** was also thermally deposited on the OTS modified substrate at a *T*_s_ of 140 °C to investigate the impact of chiral compositions on the electrical properties. Although the organic thin films were fabricated in identical conditions, **3a-*rac*** films did not show any field-effect characteristics (Supplementary Fig. 16) due to the island-type film growth (Supplementary Fig. 17).

### NIR light responsivity

**Fig. 5:**
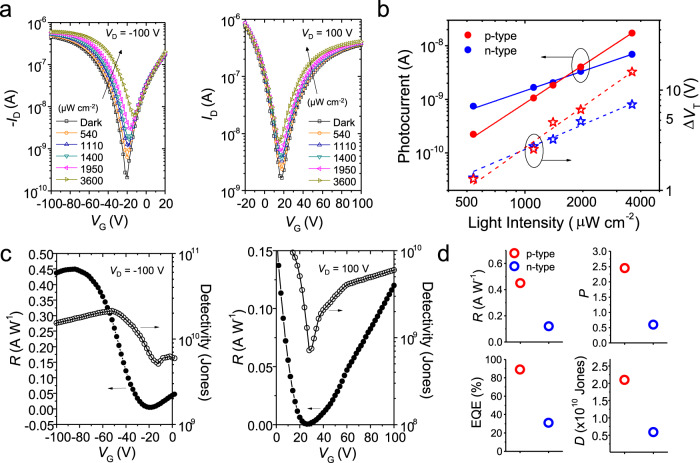
Optoelectronic performances in the dark and under NIR light irradiations. **a** Transfer curves of **3a-*P***-based OFETs in the dark and under light irradiations (*λ* = 730 nm) in *p*-type (left) and *n*-type (right) modes. **b** Quantitative analysis results of the photocurrent and *V*_T_ change of **3a-*P***-based OFETs under the NIR light irradiations. **c** *R* and D* of **3a-*P***-based OFETs under the NIR light irradiations in *p*-type (left) and *n*-type (right) modes. **d** Maximum *R*, *P*, EQE, and D* values of **3a-*P***-based OFETs under the NIR light irradiations (*λ* = 730 nm, 540 μW cm^−2^).

Design of NIR photodetector with both high quantum yield and excellent charge transport is challenging due to low dissociation efficiencies of the photogenerated excitons and a high dark current^57,58^. Notably, our novel approach using double helicene structure through *ortho*-π-extension engineering in large PDI core induces significantly red-shifted absorption capability up to NIR region and narrow band gaps. To investigate their spectral photoresponse, we measured the photocurrent of phototransistor devices as the exposed light wavelength changed from NIR to UV range. They showed photocurrent up to NIR region due to their extended π-conjugation (Supplementary Fig. 18). To further explore the photoresponsivity of the enantiopure transistors at the NIR region, the responses of **3a-*P*** to NIR light irradiations were studied (considering their CD spectra, photocurrent, and available laser, *λ* of 730 nm was selected). Significant current enhancements and threshold voltage (*V*_T_) shifts were observed in both *p*- and *n*-type operating transfer curves when an external NIR light was illuminated on **3a-*P***-based OFETs (Fig. 5a). The photocurrent enhancement and sizeable *V*_T_ shift should be attributed to the generation of photoexcited charge carriers and elimination of trap sites^59,60^. Thus, the photocurrent and *V*_T_ shift became greater with increased NIR light intensity, as shown in Fig. 5b. More importantly, the slope of photocurrent and *V*_T_ shift was steeper in *p*-type mode, indicating improved photosensing responsivity. The higher responsivity is likely originating from the enhanced electrical mobility, which could lead to easier exciton dissociation, exciton separation, and charge transport^59,61^.

The NIR detection ability was quantified by the photoresponsivity (*R*), photocurrent/dark current ratio (*P*), EQE, and detectivity (D*) parameters from the transfer characteristics in Fig. 5a. The *R* and D* values, the most important factors for photodetectors, increased with the gate field due to the efficient exciton separation within the dielectric layer (Fig. 5c). Especially, *R* values reached to 450 and 120 mA W^−1^ at *V*_G_ of −80 V and 100 V in *p*- and *n*-type modes, respectively, which are even superior to the reported chiral NIR light detectors based on silicon and chiral plasmonic-patterned electrodes (1.5 mA W^−1^)^28^. The maximum D* values of **3a-*P***-based OFETs, evaluated from the shot noise under dark conditions^60^ were estimated to be 2.1 × 10^10^ Jones and 5.9 × 10^9^ Jones, in *p*- and *n*-type operations, respectively. The maximum photoresponsive parameters under the NIR light irradiations (*λ* = 730 nm, 540 μW cm^−2^) are shown in Supplementary Table 4 and Fig. 5d. The maximum *P* of 2.5 was observed under 540 μW cm^−2^ NIR light illumination. In addition, EQE values, the ratio of the number of photogenerated carriers circulating in the circuit to the number of photons absorbed in the device, reached almost 89%, which is comparable to organic thin-film-based CPL detectors reported to date^17,26,27^.

### Selective detection of CPL

**Fig. 6:**
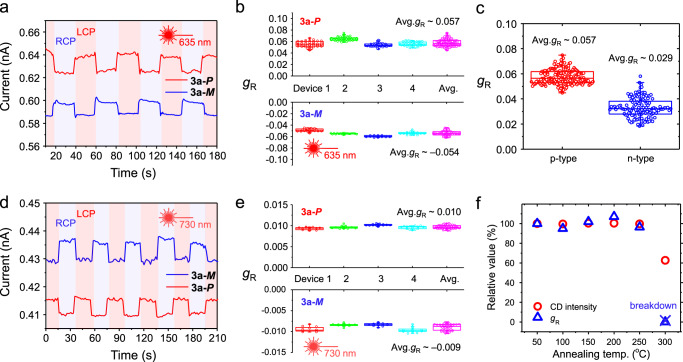
Selective CPL detection properties. **a** Real-time current signal of **3a-*P***/***M***-based OFETs under CPL irradiations (*λ* = 635 nm) in *p*-type mode (the recorded current was plotted as absolute value). **b** Quantitative analysis results of *g*_R_ of four **3a-*P***/***M***-based OFET devices under CPL irradiations (*λ* = 635 nm) in *p*-type mode. **c** Quantitative analysis results of *g*_R_ of four **3a-*P*-**based OFET devices under CPL irradiations (*λ* = 635 nm). **d** Real-time current signal of **3a-*P***/***M***-based OFETs under CPL irradiations (*λ* = 730 nm) in *p*-type mode (the recorded current was plotted as absolute value). **e** Quantitative analysis results of *g*_R_ of four **3a-*P***/***M***-based OFET devices under CPL irradiations (*λ* = 730 nm) in *p*-type mode. **f** Relative CD intensity and *g*_R_ value of **3a-*M***-based OFETs under CPL irradiations (*λ* = 635 nm) in *p*-type mode after thermal annealing.

The manipulation of NIR polarization has scarcely been explored because the conventional organic chiral semiconductors have been limited to CPL detection in UV–vis spectral region. Finally, we tested the selectivity of **3a-*P***/***M***-based OFETs for polarization of illuminated CPL by recording the real-time photocurrent signal in both *p*-type and *n*-type modes, respectively (*p*-type: *V*_D_ = −80 V, *V*_G_ = −40 V and *n*-type: *V*_D_ = 80 V, *V*_G_ = 40 V). Interestingly, **3a-*P***/***M***-based OFETs showed distinct real-time photocurrent signal under the irradiation of CPL with different polarization (*λ* = 635 nm, 1400 μW cm^−2^, Fig. 6a). **3a-*P-***based OFETs exhibited higher photocurrent under the left-handed CPL (LCPL) illumination, while **3a-*M***-based OFETs exhibited higher photocurrent under the right-handed CPL (RCPL), agreeing very well with the results of CD spectra. To investigate the CPL polarization selectivity quantitatively, we defined a dissymmetry factor of responsivity, *g*_R_, for photodetectors as follows:

1 $$g_{\mathrm{R}} = 2\left( {R_{\mathrm{{LCPL}}} - R_{{\mathrm{{RCPL}}}}} \right)/\left( {R_{{\mathrm{{LCPL}}}} + R_{{\mathrm{{RCPL}}}}} \right),$$

where *R*_LCPL_ and *R*_RCPL_ are the responsivities under the illumination of LCPL and RCPL, respectively. Figure 6b shows the *g*_R_ values estimated from four different devices in real-time CPL detections (*λ* = 635 nm). The dissymmetry factors were estimated to be −0.054 and +0.057 on average depending on the helicity of the organic molecules. The CPL detection abilities were different from the FET operation mode. In *n*-type operation, the *g*_R_ value was estimated to +0.029 in **3a-*P***-based OFETs. The more sensitive CPL detection ability may originate from the increased exciton dissociation and separation and faster charge transport in the *p*-type operation (Fig. 6c). The relatively larger *g*_R_ compared to *g*_abs_ may originate from the synergetic effect of the enhanced photocurrent difference from photomultiplication phenomena by the applied gate bias and the spin-dependent carrier transport/collection effect due to the optical selection rules^62,63^. In addition, we tested NIR CPL detection in real time (*λ* = 730 nm, 1400 μW cm^−2^, Fig. 6d, e). **3a-*P***/***M***-based OFETs also exhibited distinct and clear real-time photocurrent change with *g*_R_ of +0.010 and –0.009, respectively. These dissymmetry factors are relatively smaller than those under the red CPL illumination, which also match CD spectra. As a further control experiment for CPL detection, we tested **3a-*rac*** thin films deposited at room temperature. We tested the CPL detection in identical light exposure condition to enantiomeric devices. We recorded the current under the exposure of different handedness of CPL (635 nm). The results showed almost identical photocurrent regardless of the handedness of circular polarization within several times of exposure cycles in two devices (Supplementary Fig. 19). For practical electronic application, we also investigated the thermal stability of **3a-*P***/***M***-based OFETs. The CD intensity and *g*_R_ value under the CPL illumination keep constant even after annealing at 250 °C for 10 min (Fig. 6f). After 300 °C annealing, CD intensity sharply decreased and the OFET devices became dysfunctional because of the thermal evaporation of many domains in thin films, but was not induced by the isomerization process. These stable and unique NIR light detection should make the helical semiconductors extremely valuable in the high-performance diverse chiroptical applications.

## Discussion

In conclusion, we demonstrated a skeleton merging approach for the design of PDI double-heterohelicenes as chiral organic semiconductors for broadband CPL detections. The resulting tetraindole-fused PDI **3a** offered a long-desired, simultaneous achievement of electrical and optical properties with large circularly polarization selectivity. The *ortho*-π-extended PDI double helicene with high photoresponsivity and detectivity enabled CPL detection at NIR spectral region for the first time among chiral organic semiconductors, together with ambipolar charge transport and excellent thermal stability up to ~250 °C. Furthermore, the high-performance NIR-responsive, ambipolar chiral organic semiconductors with intrinsically high *g*_abs_ developed in this study could be utilized for the fabrication of integrated CPL detectors and complementary logic circuits without auxiliary optical elements. Thus, the skeleton merging approach provides opportunities for the design of custom-made molecules to pursue sophisticated multiple characteristics in order to meet the demands of miniaturization trends in the coming “Internet of Things (IoT)” era.

## Methods

All commercial reagents were used without further purification unless otherwise indicated. All oxygen-sensitive reactions were performed under argon atmosphere using the standard Schlenk method. ^1^H and ^13^C NMR spectra were recorded at 400 or 500 MHz in CDCl_3_ as solvent. High-resolution mass spectra were obtained on an FT-MS instrument using ESI or APCI technique. Absorption spectra were recorded on a Thermo Scientific Evolution 300 UV/Vis spectrophotometer. CD spectra of solutions were recorded with a JASCO J-1500 spectropolarimeter. Absorption and CD spectra of thin films were recorded with a JASCO V-770 and a JASCO-815 150-L with an equipment for solid samples, respectively. For transmission spectroscopy, we thermally deposited thin films on transparent quartz plates. Cyclic voltammetry measurements were performed on a Bio-Logic-Science Instrument EC-LAB SP-200. The *g*_abs_ values are typically defined by the following equation:

2 $$g_{{\mathrm{abs}}} = \frac{\Theta }{{33000 \ast {\mathrm{{Abs}}}}}.$$

*DFT calculations* were performed using the Gaussian 09 software package. The geometries and frequencies calculations have been obtained with the ωB97Xd functional in combination with the 6–31+G(d) basis set. Alkyl chains at imide position have been truncated to methyl groups to simplify the calculations. For calculations of CD spectra, the first 20 states were calculated for the [7]-heterohelicenes molecules and the first 40 states for the PDI double-heterohelicenes. A Gaussian broadening of 0.2 eV was used.

### OFET fabrication

OFETs based on **3a** and **3b** were fabricated using heavily doped silicon wafers covered with a 300-nm-thick SiO_2_ layer (*C*_*i*_ = 11.5 nF cm^−2^). Wafers were cleaned with a piranha solution for 30 min, followed by UV-ozone treatment. The wafer surface was treated with an OTS self-assembled monolayer. The OTS solution (3 mM in trichloroethylene) was spin-coated onto the wafers at 1500 r.p.m. for 30 s, and the samples then kept overnight in a vacuum desiccator with a separate vial containing NH_4_OH. The wafers were then washed with toluene, acetone, and isopropyl alcohol and dried under nitrogen. To fabricate thin films, PDI derivatives were deposited (40 nm) onto OTS-treated wafers in a thermal evaporator at different substrate temperatures. The chamber was under high vacuum (<5.0 × 10^−6^ torr), and the deposition rate was maintained at 0.2 Å s^−1^. Gold electrodes (40 nm) were thermally evaporated and patterned using shadow masks. The source/drain patterns had a channel length (*L*) of 50 μm and a channel width (*W*) of 1000 μm (*W*/*L* = 20).

### Optoelectronic measurements

Current–voltage characteristics of OFETs were measured inside a vacuum chamber, using a Keithley 4200-SCS semiconductor parametric analyzer. To investigate photocurrent of phototransistor devices, laser instrument (CNI laser, maximum power of 5 mW) was used to generate monochromatic light. For testing the spectral photoresponse, monochromatic light was produced using a 300 W Xenon lamp and Oriel Cornerstone 130 monochromator with dual gratings. In all, 280 μm slits were used for bandwidth of 3.7 nm. The CPL illumination was generated through a linear polarizer and a quarter-wave plate (Thorlabs). To confirm the quality of circular polarization of the light, we tested the intensity independence of the light passing through the quartz as the degree between the transmission axis of the linear polarizer and the fast axis of the quarter-wave plate changed. Also, we rotated the degree to avoid elliptical polarization by confirming that the intensity of light splits into two orthogonal linear polarization state using beam splitter (Thorlabs). All corrections were conducted by placing the Si photodetector in the same position with the samples.

### Estimation of optoelectrical properties

In order to investigate photosensitivity for OFETs, photoresponsivity (*R*) and photocurrent/dark current ratio (*P*) were calculated from transfer characteristics coupled with light irradiation. The *R* and *P* values are typically defined by the following equations:

3 $$R = \frac{{I_{{\mathrm{ph}}}}}{{P_{{\mathrm{inc}}}}} = \frac{{I_{{\mathrm{light}}} - I_{{\mathrm{dark}}}}}{{P_{{\mathrm{inc}}}}},$$

4 $$P = \frac{{I_{{\mathrm{light}}} - I_{{\mathrm{dark}}}}}{{I_{{\mathrm{dark}}}}},$$

where *I*_ph_ is the photocurrent, *P*_inc_ the incident illumination power on the channel of the device, *I*_light_ the drain current under illumination, and *I*_dark_ the drain current in the dark, respectively. In addition, the EQE (*η*) of OPTs was calculated, which can be defined as the ratio of number of photogenerated carriers that practically enhances the drain current to the number of photons incident onto the OPT channel area, using the following Eq. (5):

5 $$\eta = \frac{{\left( {I_{{\mathrm{{light}}}} - I_{{\mathrm{{dark}}}}} \right)hc}}{{eP_{{\mathrm{{int}}}}A\lambda _{{\mathrm{{peak}}}}}},$$

where *h* is the plank constant, *c* the speed of light, *e* the fundamental unit of charge, *P*_int_ the incident power density, *A* the area of the transistor channel, and *λ*_peak_ the peak wavelength of the incident light, respectively.

Detectivity usually describes the smallest detectable signal, which allows comparisons of phototransistor devices with different configurations and areas. D* was evaluated within this study using the following Eqs. (6) and (7):

6 $$D^ \ast = \frac{{\sqrt A }}{{{\mathrm{{NEP}}}}},$$

7 $${\mathrm{{NEP}}} = \frac{{\sqrt {\overline {I_n^2} } }}{{R\sqrt {\Delta f} }}.$$

In these equations, *A* is the phototransistor active area, NEP the noise equivalent power, $\bar I_n^2$ the measured noise current, and Δ*f* the bandwidth. If the major limit to detectivity is shot noise from the drain current under dark conditions, D* can be simplified as

8 $$D^ \ast = \frac{R}{{\sqrt {\left( {2e \cdot I_{{\mathrm{{dark}}}}/A} \right)} }}.$$

## Supplementary information

Supplementary Information

## Data Availability

The data supporting the findings of this study are included within the Article and its Supplementary Information files, and are also available from the authors upon reasonable request.
