# Supplementary material for: π-Extended perylene diimide double-heterohelicenes as ambipolar organic semiconductors for broadband circularly polarized light detection
Source: Nat Commun. 2021 Jan 8;12:142. doi: 10.1038/s41467-020-20390-y (PMC7794514; doi:10.1038/s41467-020-20390-y)
Supplement: Supplementary file 1 — Supplementary Information [file 41467_2020_20390_MOESM1_ESM.pdf]

Supplementary Information for

π-Extended Perylene Diimide Double-Heterohelicenes as Ambipolar Organic Semiconductors for Broadband Circularly Polarized Light Detection

Li Zhang[1,6], Inho Song[2,6], Jaeyong Ahn[2], Myeonggeun Han[3], Mathieu Linares[4], Mathieu Surin[5], Hui-Jun Zhang[1], Joon Hak Oh[2]✉ & Jianbin Lin[1]✉

[1]Department of Chemistry, College of Chemistry and Chemical Engineering, MOE Key Laboratory of Spectrochemical Analysis and Instrumentation, Xiamen University, Xiamen 361005, P. R. China.
[2]School of Chemical and Biological Engineering, Institute of Chemical Processes, Seoul National University, 1 Gwanak-ro, Gwanak-gu, Seoul 08826, Korea.
[3]Department of Chemical Engineering, Pohang University of Science and Technology (POSTECH), Gyeongbuk, Pohang 37673, Korea.
[4]Laboratory of Organic Electronics and Scientific Visualization Group, ITN, Campus Norrköping; Swedish e-Science Research Centre (SeRC), Linköping University, SE-581 83, Sweden.
[5]Laboratory for Chemistry of Novel Materials, Centre of Innovation and Research in Materials and Polymers (CIRMAP), University of Mons - UMONS, 20 Place du Parc, Mons B-7000, Belgium.
[6]These authors contributed equally to this work.
✉email: joonhoh@snu.ac.kr; jb.lin@xmu.edu.cn.

Supplementary Figures

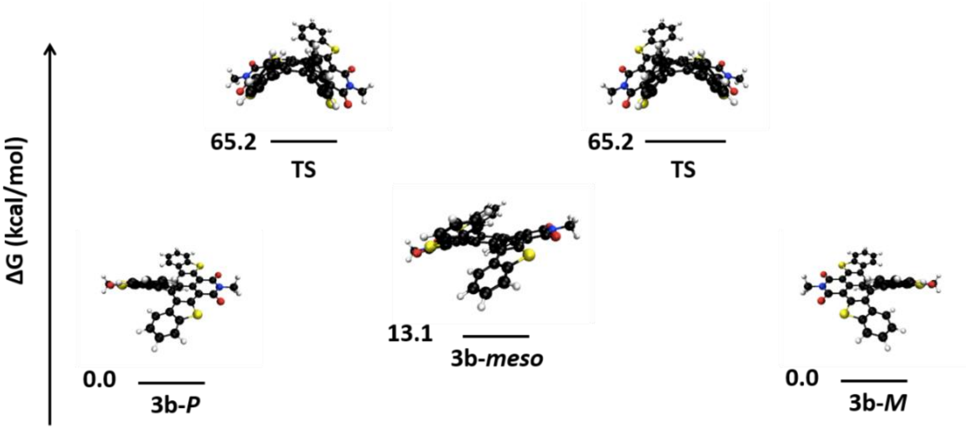

Supplementary Figure 1. **Potential energy surface of the isomerization process between 3b-*P*/*M* and 3b-*meso* conformations.** Calculated by DFT calculations at the ωB97Xd/6-3+1G(d) level (hexylheptyl groups have been replaced by methyl groups to simplify the calculations, Carbon = black, Hydrogen = white, Nitrogen = blue, Oxygen = red.).

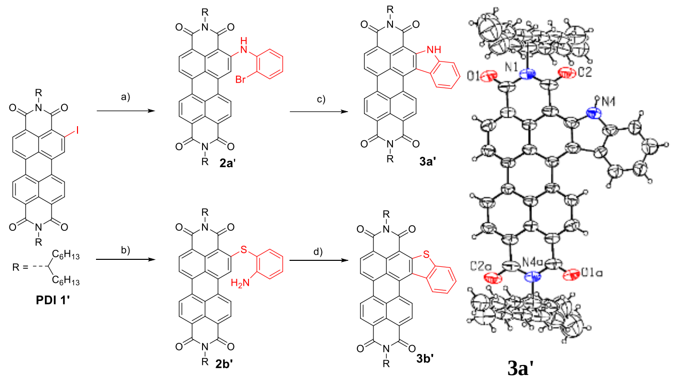

Supplementary Figure 2. **Synthesis of *ortho*-mono-indole and *ortho*-mono-benzothiophene fused PDIs 3a' and 3b'**. Reagents and conditions: a) **2a'**: 2-bromoaniline, ^{t}BuOK, 120 °C, 2.5 h, yield 73%; b) **2b'**: 2-aminobenzenethiol, K_2CO_3, NMP, 120 °C, 3 h, yield 72%; c) **3a'**: $Pd(OAc)_2$, K_2CO_3, dimethylformamide (DMF), 140 °C , 12 h, yield 77%; d) **3b'**: isoamyl nitrite, DCM/HOAc, Cu_2O, 0 °C, yield 82%.

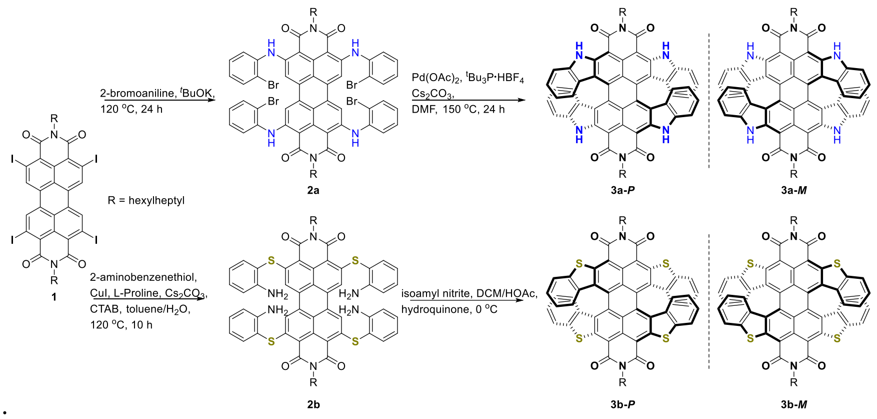

Supplementary Figure 3. **Synthesis of *ortho*-tetra-indole and *ortho*-tetra-benzothiophene fused PDIs 3a and 3b**.

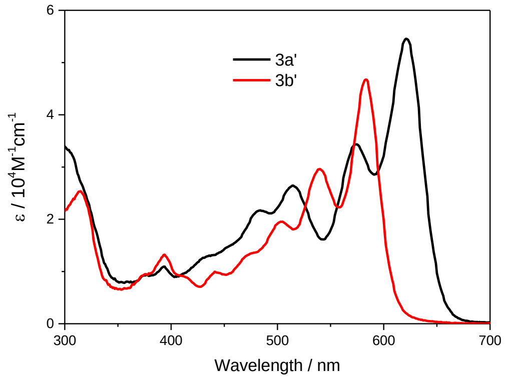

Supplementary Figure 4. UV-visible absorption spectrum of **3a', 3b'**. (10 μM in chloroform).

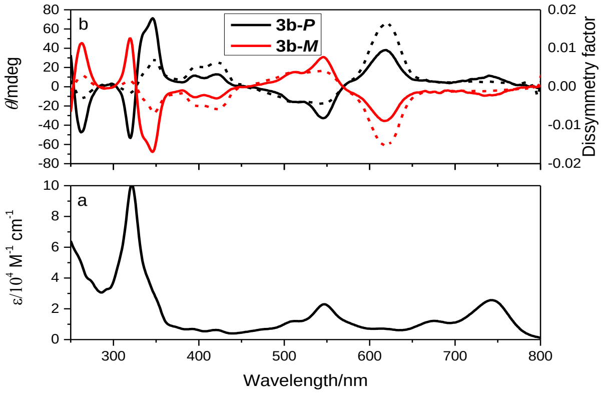

Supplementary Figure 5. a) **UV-visible absorption** and b) **CD spectrum/dissymmetry factor (g_{abs})** of **3b** (10 μM in chloroform, solid line: CD, dashed line: g_{abs}).

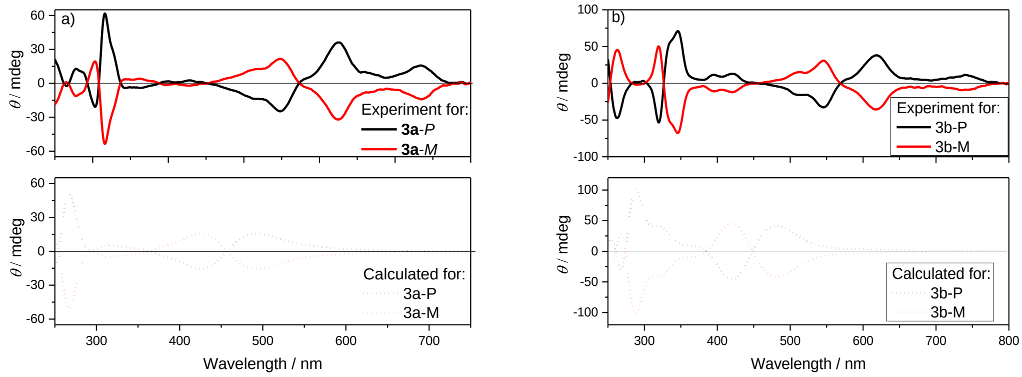

Supplementary Figure 6. **Assignment of the absolute configuration** of a) **3a** and b) **3b** by comparison of their experiment CD (solid lines) with the calculated spectra (dotted lines). (A shift of wavelengths between calculated and experimental CD spectra is typical from such calculations, and can be explained for instance by the fact that solvent effect and vibronic contribution are not included in the calculations.)

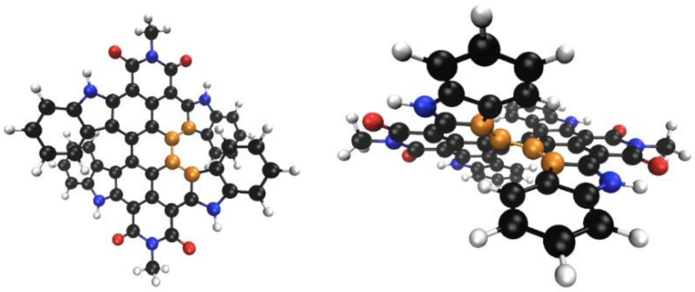

Supplementary Figure 7. **Twist of the PDI moiety, the atoms used for the calculation of the twist are highlighted in orange.** Top view (Left) and Side view (right) (The first band at low energy can be attributed to a deformation of the PDI core of the molecule from a planar conformation with a twist of +33.6° and -33.6° for the **3a-*P*** and **3a-*M*** conformers, respectively, and +35.8° and -35.8° for the **3b-*P*** and **3b-*M*** conformers, respectively.)

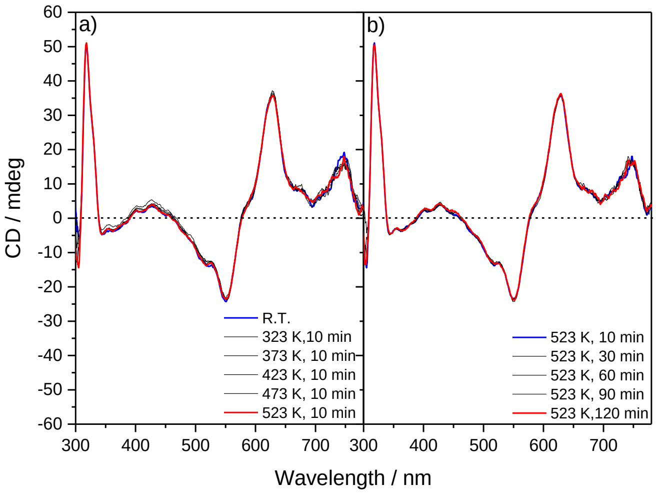

Supplementary Figure 8. a) **Temperature-dependent** (heating 10 mins before measurement), b) **Time-dependent** (at 523 K) **CD spectrum** of **3a-*P*** in diphenyl ether (10 µM).

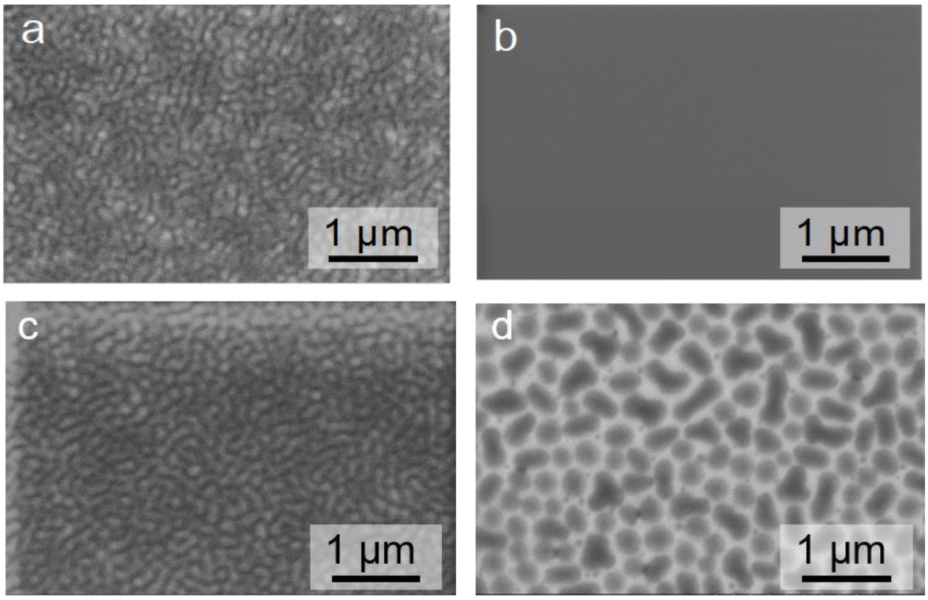

Supplementary Figure 9. **SEM images** of films deposited at an optimized substrate temperature of a) **3a-*P*** (140 °C), b) **3a-*P*** (RT), c) **-*M*** (140 °C), and d) **-*rac*** (140 °C).

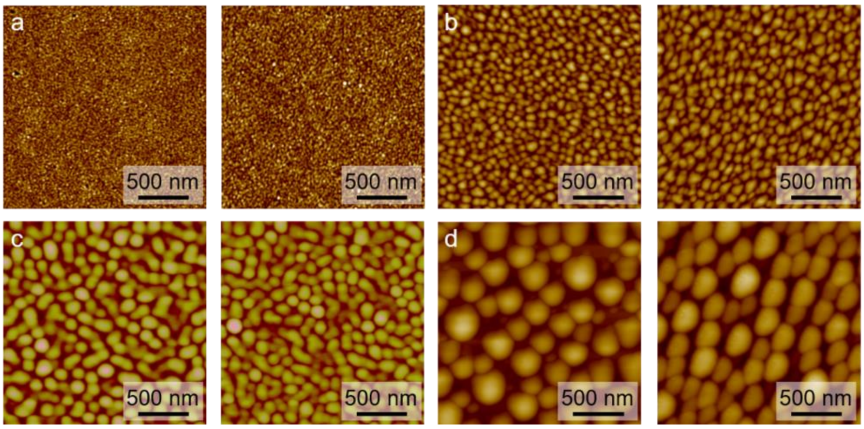

Supplementary Figure 10. **AFM topography images** of **3a-*P/M*** films deposited at substrate temperature of a) RT, b) 80 °C, c) 140 °C, and d) 200 °C (left: **3a-*P*** right: **3a-*M***).

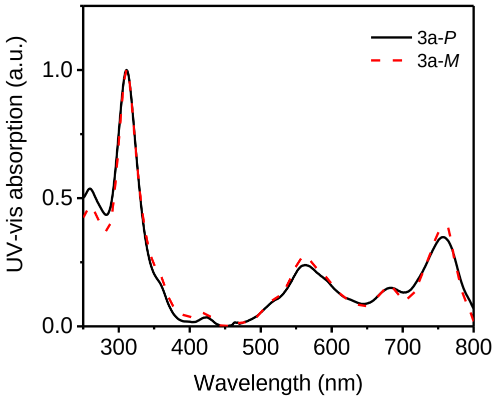

Supplementary Figure 11. **UV-vis absorption spectra** of **3a-*P*/*M*** films deposited at an optimized substrate temperature of 140 °C.

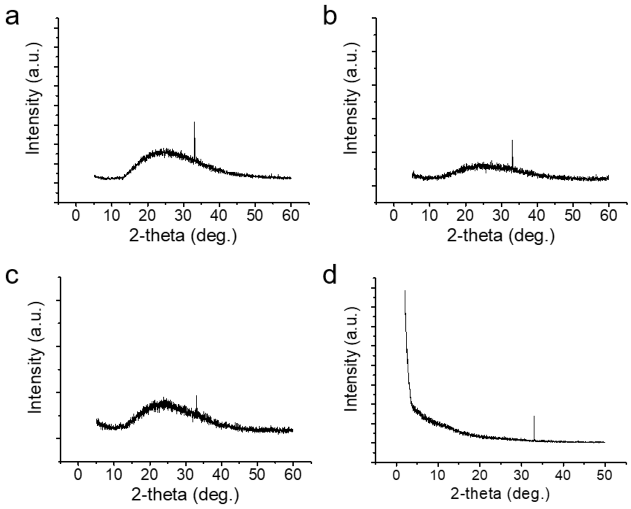

Supplementary Figure 12. **PXRD results** of a) **3a-*P*** and b) **-*M*** films deposited at an optimized substrate temperature of 140 °C. PXRD results of c) **3a-*P*** and b) **-*rac*** films deposited at a substrate temperature of RT.

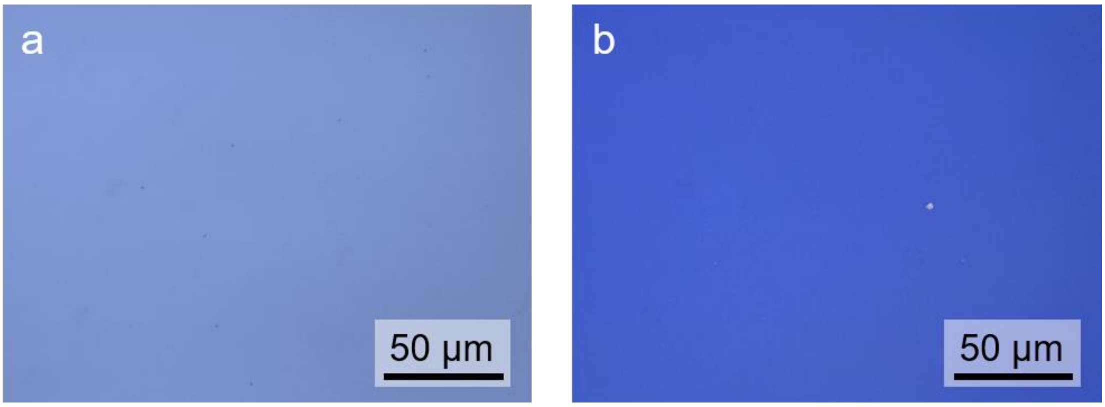

Supplementary Figure 13. Optical images of a) **3a-*P*** and b) **-*M*** films deposited at optimized substrate temperature of 140 °C.

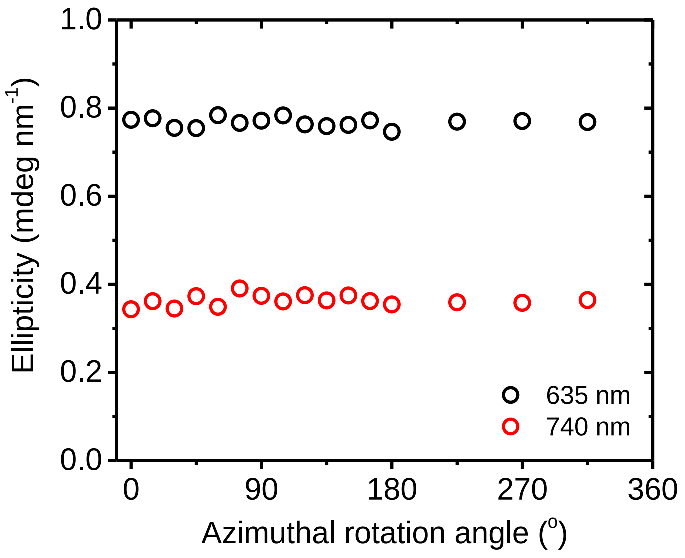

Supplementary Figure 14. **Recorded thickness normalized ellipticity values** of **3a-*P*** thin films deposited at 140 °C depending on the azimuthal sample rotation angle at wavelength of 635 nm and 740 nm.

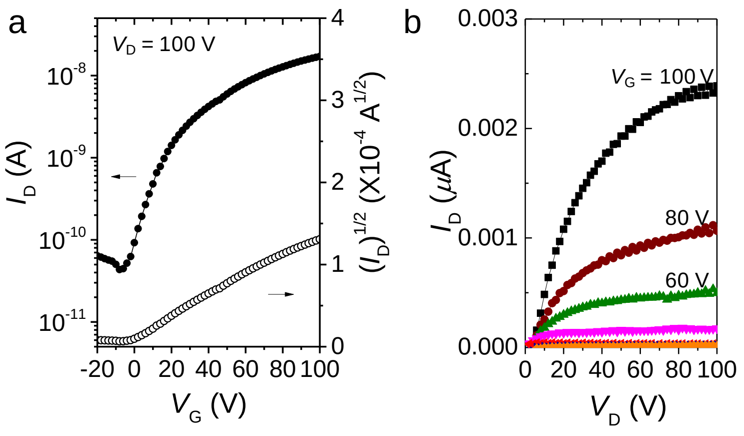

Supplementary Figure 15. a) **Transfer characteristics** of OFETs based on **3b-*P*** films. b) **Output characteristics** of OFETs based on **3b-*P*** films.

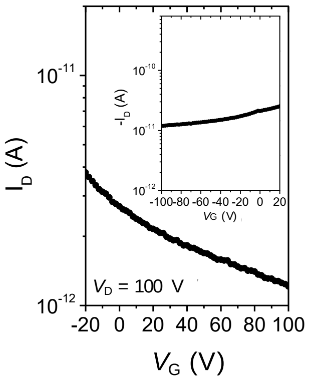

Supplementary Figure 16. **Transfer characteristics** of OFETs based on **3a-*rac*** films deposited at substrate temperature of 140 °C.

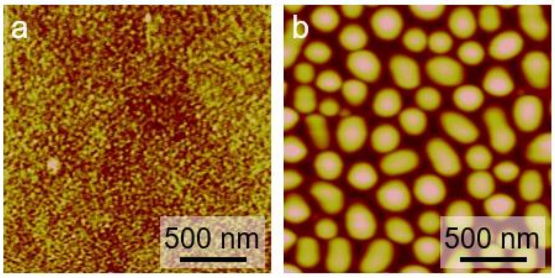

Supplementary Figure 17. AFM topography images of **3a-*rac*** films deposited at substrate temperature of a) RT and b) 140 ºC. (AFM measurement of both **3a-*P*** and **3a-*rac*** films deposited at a T_s of 140 °C revealed that thin films of **3a-*P*** consisted of large, but dense granular crystalline domains. However, **3a-*rac*** films exhibited very bulky, island-type aggregated domains on OTS surface. Quantitative analysis on the root-mean-square roughness (R_{rms}) values and height profiles along the red lines in AFM height images disclosed that the thickness of **3a-*P*** films was not random but amounts to ~10 nm, and was much lower than that of **3a-*rac*** films (~60 nm). Scanning electron microscope (SEM) analysis on the **3a-*P*** and **3a-*rac*** films displayed similar trends to the AFM results. Overall, well-connected characteristics between domains are found to be an important factor for efficient charge transport.

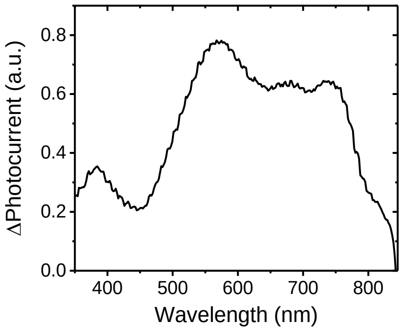

Supplementary Figure 18. **Spectral photocurrent response** of **3a-*P*** thin film based phototransistors.

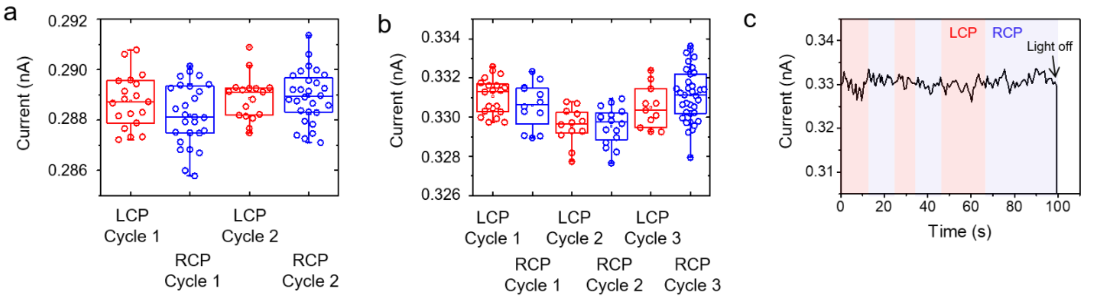

Supplementary Figure 19. a-b) Current plot under the left-handed circularly polarized (LCP) light and right-handed circularly polarized (RCP) light illuminations of **3a-*rac*** based OFETs in *p*-type mode. c) Real-time current signal of **3a-*rac*** based OFETs under CPL irradiations (λ = 635 nm) in *p*-type mode for b).

Supplementary Tables

Supplementary Table 1. Electrochemical properties of compounds **3a'-b'**, **3a-b**

PDI	E_{red1} [V][a]	E_{red2} [V][a]	E_g [eV][b]	LUMO [eV][c]	HOMO [eV][d]
**3a'**	-1.07	-1.27	1.80	-3.73	-5.53
**3b'**	-1.00	-1.2	1.90	-3.80	-5.70
**3a**	-1.05	-1.21	1.56	-3.75	-5.31
**3b**	-0.80	-0.97	1.53	-4.00	-5.53

[a]Determined by cyclic voltammetric measurement in 0.1 M solution of Bu_4NPF_6 in DCM: vs Fc/Fc^+. [b]E_g = optical gap, calculated from the optical absorption data. [c]LUMO was estimated vs vacuum level from LUMO = 4.80 eV - E_{red1}.[d]HOMO was calculated from HOMO = LUMO - E_g.

Supplementary Table 2. OFET performances based on **3a** and **3b** films at different chiral composition and substrate temperatures.

PDI	Chiral Composition	Substrate Temp. (°C)	Transistor type	I_{on} / I_{off}	V_t[a] (V)	μ[a] ($cm^2 V^{-1} s^{-1}$)
**3a**	***Enantiomer***	140	N	$>10^4$	-20.2 (3.1)	$1.7 (0.2) \times 10^{-3}$
			P	$>10^3$	-22.3 (4.6)	$2.1 (0.3) \times 10^{-3}$
		RT	N	$>10^2$	2.4 (0.3)	$2.3 (0.3) \times 10^{-5}$
			P	>10	-12.1 (1.9)	$2.8 (0.2) \times 10^{-5}$
	Racemate	140	N	-	-	-
			P	-	-	-
		RT	N	>10	15.7 (4.4)	$3.4 (0.6) \times 10^{-6}$

			P	>10	-43.4 (7.8)	$4.2 (0.7) \times 10^{-6}$
**3b**	***Enantiomer***	140	N	$>10^{2}$	-6.3 (0.7)	$2.5 (0.2) \times 10^{-5}$
			P	-	-	-

[a]The average and standard deviation values were estimated from more than 5 devices

Supplementary Table 3. Literature table for reported helical-shaped organic semiconductors.

References	CD active (nm)	μ_h ($cm^2 V^{-1} s^{-1}$)	μ_e ($cm^2 V^{-1} s^{-1}$)
Nature Photonics **2013**, 7, 634–638.	300-400	0.0001	-
ACS Nano **2017**, 11, 8329−8338	-	0.000024	-
Angew. Chem. Int. Ed. **2018**, 57, 10933–10937	300-700	-	0.26[a]
Organic Electronics **2009**,10, 1511–1520	-	0.00000017	-
J. Am. Chem. Soc. **2012**, 134, 19600−19603	250-300	0.00079[b]	0.0045[b]
Adv. Funct. Mater. **2015**, 25, 5640–5649	400-600	Almost no mobility[c]	-
Adv. Funct. Mater. **2014**, 24, 4943−4951	300-600	0.00022	-
J. Am. Chem. Soc. **2011**, 133, 8654–8661	350-600	0.00001	-
(This work) **3a-*P*/*M***	300-780	0.0021	0.0017

[a]Estimated using single crystal OFETs.
[b]Estimated from time-of-flight (TOF) method, not using OFETs.
[c]Displayed transistor characteristics, but hard to estimate charge mobility due to poor electrical performance.

Supplementary Table 4. Photoparameters based on **3a-*P*** films under the light illumination (λ = 730 nm, 540 μW cm^{-2}).

**Transistor type**	I_{ph} **(A)**	V_t **(V)**	***R*** **(A W^{-1})**	***P***	**EQE (%)**	D^* **(Jones)**
**P**	7.1×10^{-8}	1.3	0.45	2.45	89	2.1×10^{10}
**N**	1.5×10^{-8}	1.3	0.12	0.60	31	5.9×10^{9}

Supplementary Notes

Supplementary Note 1

Synthesis of 2a'. To a 15-mL Schlenk tube, PDI **1'**[1](0.2 mmol, 176.2 mg), ^{t}BuOK (0.3 mmol, 33.7 mg) and *o*-bromoaniline (0.5 mL) were added under argon. Then, the tube was sealed and the mixture was stirred at 120 ℃ for 2.5 hours. After cooling down to ambient temperature, 10 mL of methanol was added and precipitation was collected by filtration, then, the precipitation was purified by silica gel column chromatography using DCM/hexane as eluent to afford the red product **2a'**. (135.1 mg, 73%)

2a': ^{1}H NMR (500 MHz, $CDCl_3$) δ 11.86 (d, *J* = 15.6 Hz, 1H), 8.70 – 8.51 (m, 4H), 8.38 (d, *J* = 8.2 Hz, 1H), 8.27 – 8.14 (m, 2H), 7.83 (d, *J* = 8.0 Hz, 1H), 7.61 (d, *J* = 7.5 Hz, 1H), 7.51 (t, *J* = 7.6 Hz, 1H), 7.30 – 7.26 (m, 1H), 5.29 (ddd, *J* = 9.1, 6.0, 3.2 Hz, 1H), 5.21 – 5.12 (m, 1H), 2.33 – 2.18 (m, 4H), 1.93 – 1.81 (m, 4H), 1.34 – 1.21 (m, 32H), 0.82 (q, *J* = 7.0 Hz, 12H). ^{13}C NMR (101 MHz, $CDCl_3$) δ 167.5, 167.1, 164.7, 163.6, 150.1, 137.7, 135.4, 135.2, 134.9, 134.4, 134.2, 133.9, 132.0, 131.7, 129.4, 128.7, 127.6, 126.4, 126.0, 123.1, 122.9, 120.7, 119.6, 112.3, 101.5, 101.1, 54.8, 32.5, 31.92, 31.88, 29.4, 29.3, 27.13, 27.05, 22.8, 22.7, 14.20, 14.16. HRMS(APCI) calcd for $C_{56}H_{67}BrN_3O_4^+$ [M + H]$^+$: 924.4309; Found: 924.4304.

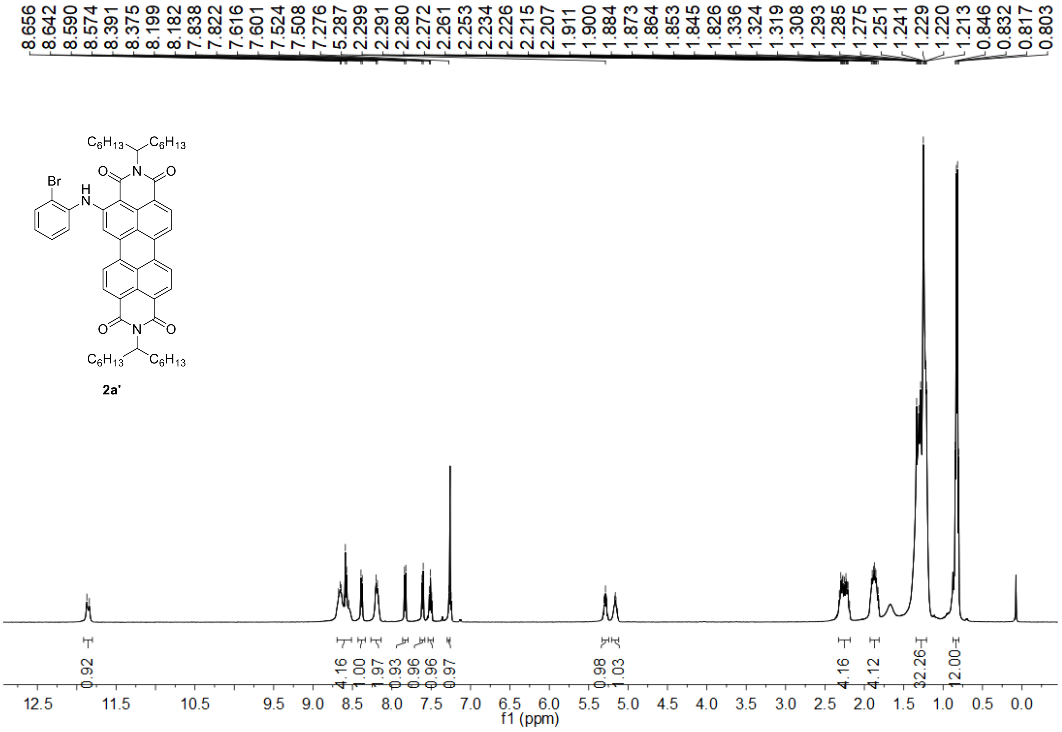

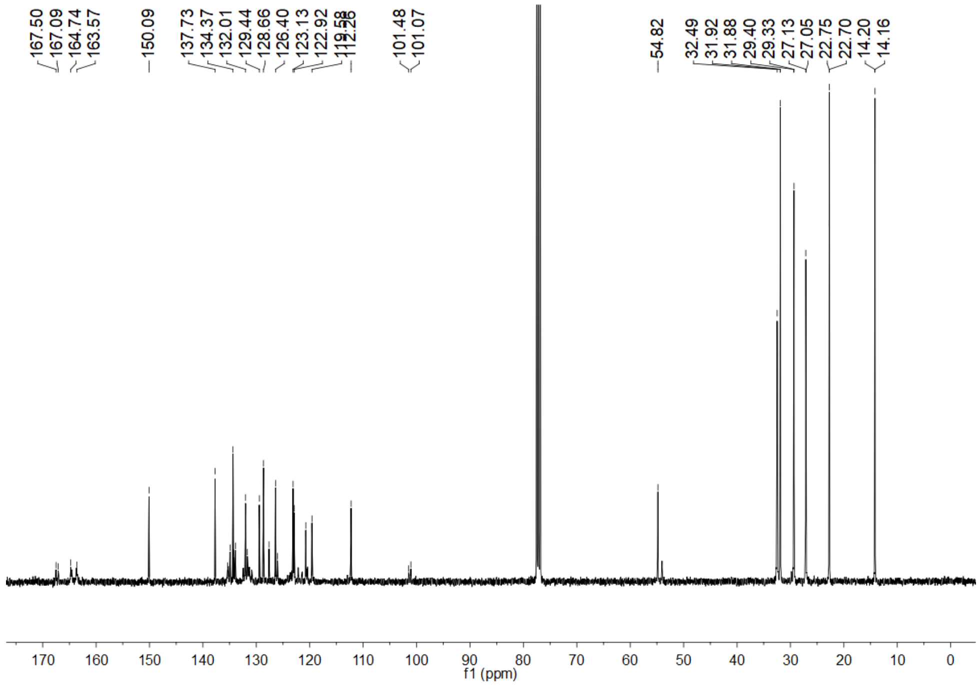

Synthesis of 2b'. To a 15-mL Schlenk tube, PDI **1'** (0.1 mmol, 88.1 mg), K_2CO_3 (0.05 mmol, 7.0 mg) were added under argon, then *o*-aminothiophenol (0.11 mmol, 11.8 uL) and NMP (1mL) were injected, the tube was sealed and the mixture was stirred at 120 ℃ for 3 hours. After the reaction cooled down to room temperature, 10 mL of water was added, the mixture was extracted by DCM, then, the organic phase was washed with brine and dried with Na_2SO_4. After removal of the solvent, the resident solid was purified by silica gel column chromatography using DCM/hexane as eluent, finally the red product **2b'** was obtained. (63.2 mg, 72%)

2b': ^{1}H NMR (500 MHz, $CDCl_3$) δ 8.71 – 8.43 (m, 5H), 7.91 (s, 1H), 7.77 (d, J = 7.8 Hz, 1H), 7.61 (d, J = 7.4 Hz, 1H), 7.51 (t, J = 6.9 Hz, 1H), 7.14 – 6.93 (m, 2H), 5.30 – 5.21 (m, 1H), 5.18 – 5.09 (m, 1H), 2.94 (s, 2H), 2.31 – 2.16 (m, 4H), 1.98 – 1.90 (m, 2H), 1.87 – 1.79 (m, 2H), 1.34 – 1.18 (m, 32H), 0.85 – 0.80 (m, 12H). ^{13}C NMR (126 MHz, $CDCl_3$) δ 164.6, 163.6, 149.5, 138.0, 134.6, 134.4, 134.3, 133.1, 132.8, 132.0, 131.1, 129.5, 126.6, 124.2, 123.12, 123.07, 121.9, 121.9,116.2, 114.1, 54.9, 32.6, 32.5, 31.94, 31.89, 29.4, 29.3, 27.2, 27.1, 22.8, 22.7, 14.2, 14.1. HRMS (ESI) calcd for $C_{56}H_{67}N_3O_4SNa^+$ [M + Na]$^+$: 900.4744; Found: 900.4741.

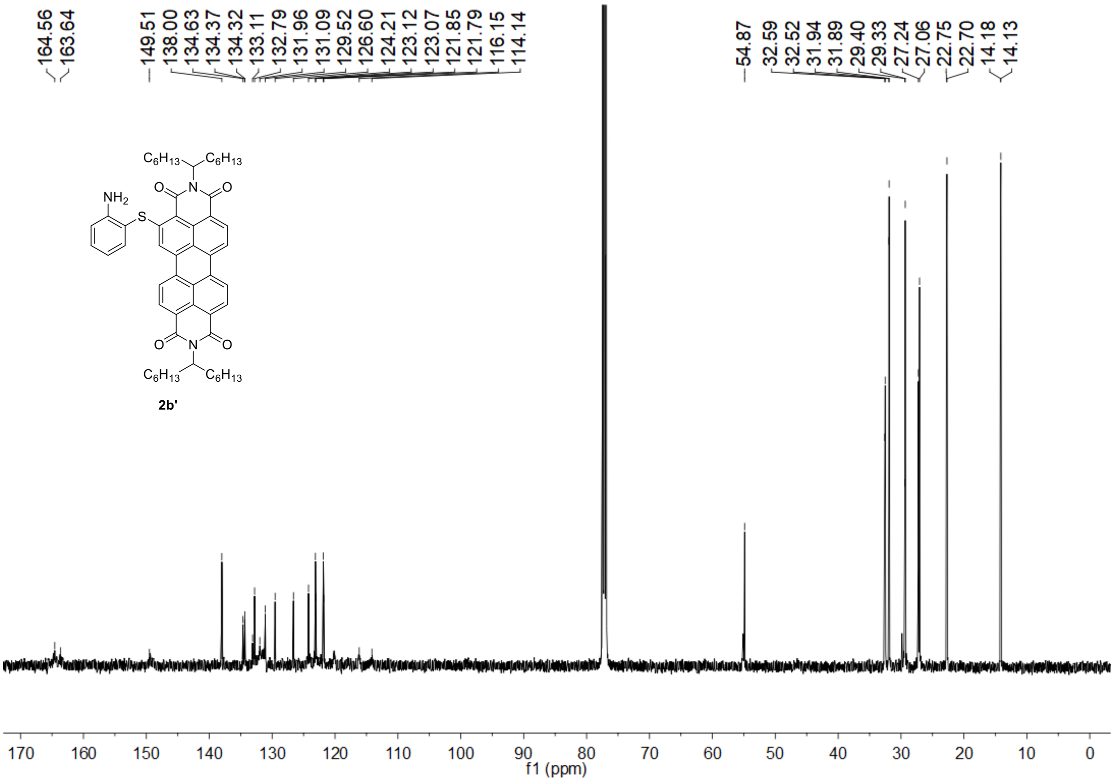

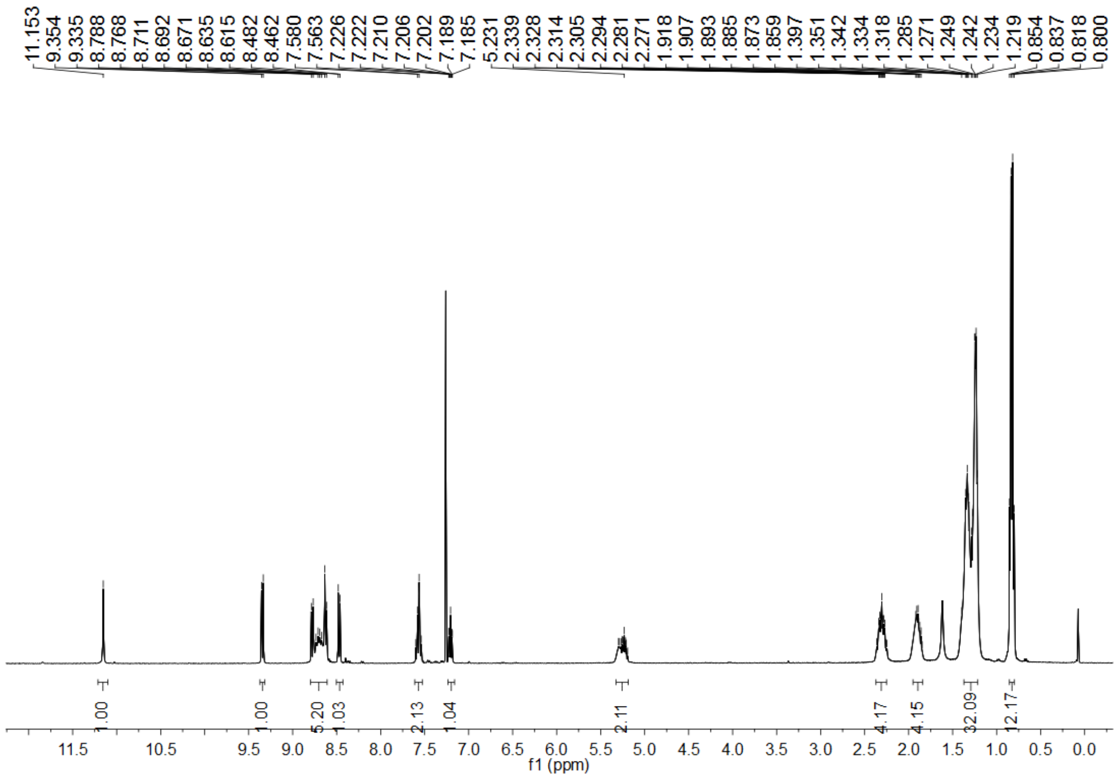

Synthesis of 3a'. To a 15-mL Schlenk tube, **2a'** (0.05 mmol, 46.2 mg), $Pd(OAc)_2$ (20 mol%, 0.01 mmol, 2.2 mg), K_2CO_3 (0.1 mmol, 13.8 mg) and dry DMF (2 mL) were added under argon. The mixture was stirred in 140 ℃ oil bath overnight. After the reaction was cooled down to room temperature, 10 mL of water was added, the mixture was extracted by DCM, then, the organic phase was washed with brine and dried with Na_2SO_4. After removal of the solvent, the resident solid was purified by silica gel column chromatography using DCM/hexane as eluent, finally the mazarine product **3a'** was obtained. (32.4 mg, 76.8%)

3a': ^1H NMR (400 MHz, $CDCl_3$) δ 11.15 (s, 1H), 9.34 (d, J = 7.9 Hz, 1H), 8.80 – 8.61 (m, 5H), 8.47 (d, J = 8.2 Hz, 1H), 7.61 – 7.53 (m, 2H), 7.24 – 7.16 (m, 1H), 5.32 – 5.19 (m, 2H), 2.37 – 2.25 (m, 4H), 1.95 – 1.84 (m, 4H), 1.37 – 1.21 (m, 32H), 0.83 (q, J = 7.0 Hz, 12H). ^{13}C NMR (126 MHz, $CDCl_3$) δ 166.6, 166.1, 164.9, 164.1, 144.6, 143.4, 135.0, 134.7, 134.6, 132.0, 130.8, 130.2, 129.2, 128.4, 127.8, 127.2, 125.1, 123.2, 122.4, 122.01, 121.95, 120.69, 120.67, 112.3, 102.0, 101.4, 55.0, 32.6, 31.9, 29.4, 27.22, 27.15, 22.8, 22.7, 14.16, 14.15. HRMS (APCI) calcd for $C_{56}H_{66}N_3O_4^+$ [M + H]$^+$: 844.5048; Found: 844.5041.

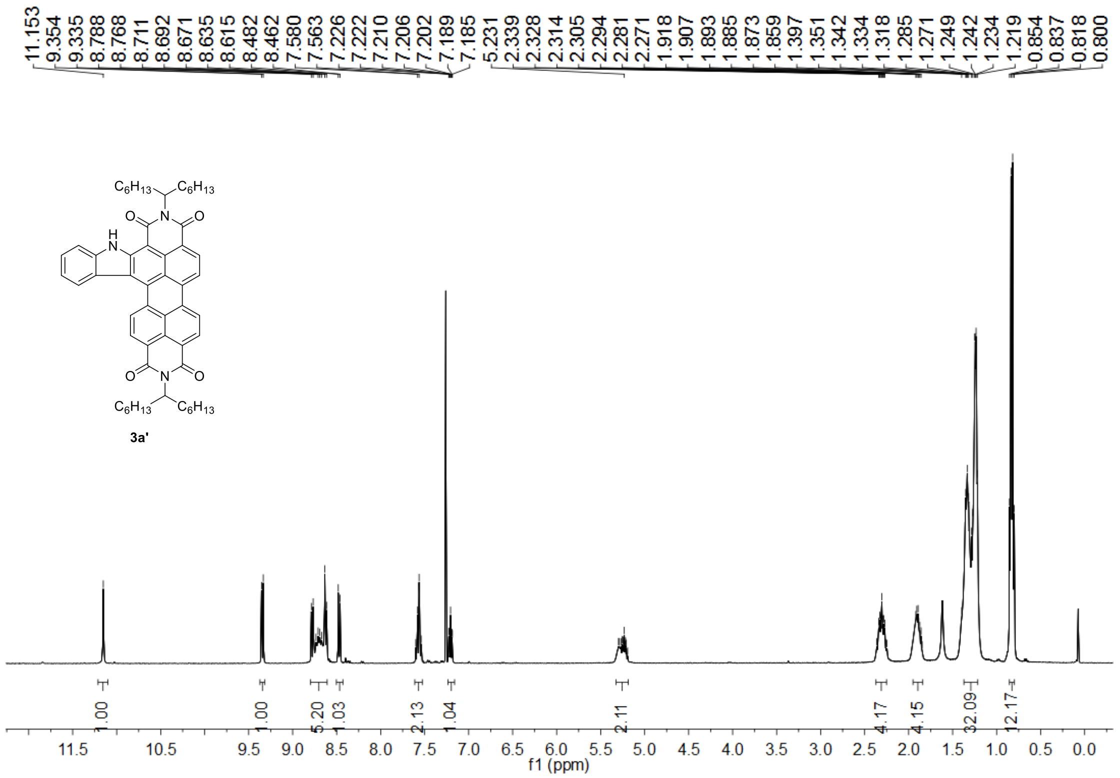

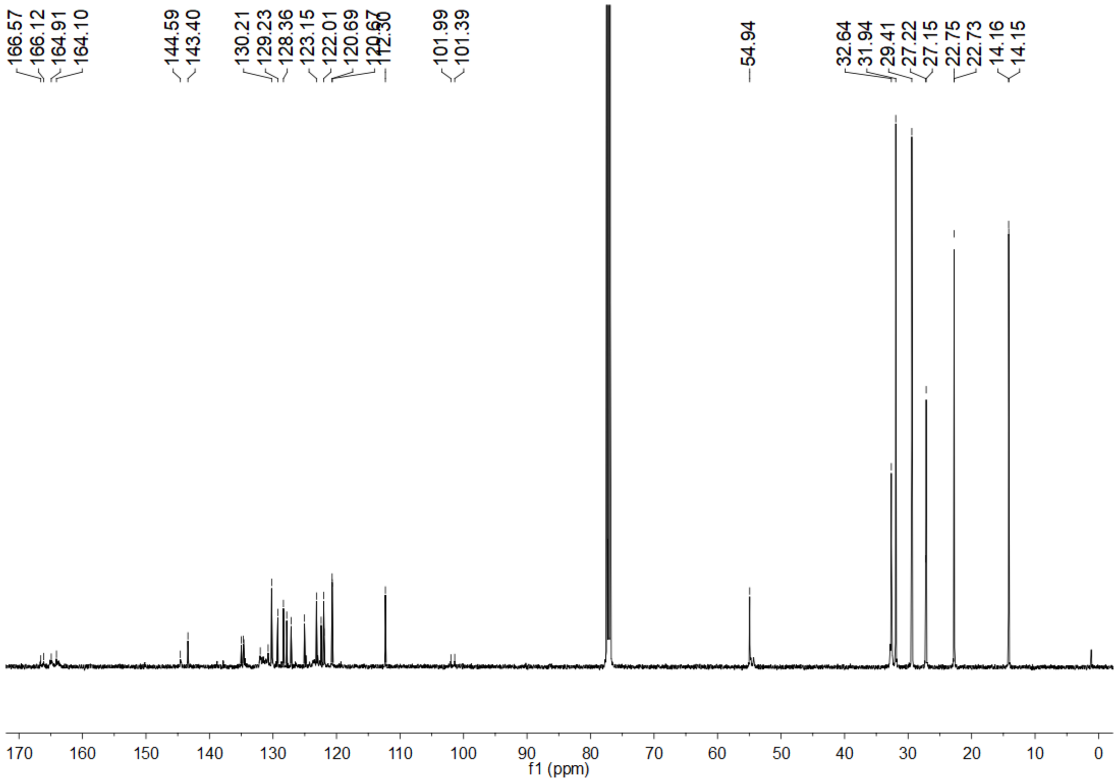

Synthesis of 3b'. Dissolve **2b'** (0.05 mmol, 43.9 mg) in a mixture of DCM (4 mL) and AcOH (4 mL), isoamyl nitrite (0.5 mmol, 67.2 uL) was injected into the solution while cooling to 0 ℃, the diazonium salt was continued to stir in the ice water bath for 30 minutes. Then Cu_2O (0.15 mmol, 21.5 mg) was added to the reaction, the mixture was warmed up to room temperature and continued to stir for additional 30 minutes. The organic solvent was removed by rotary evaporation, the violet

product **3b'** was obtained by silica gel column chromatography using DCM/hexane as eluent. (35.5 mg, 82.4%)

3b': ^{1}H NMR (500 MHz, $CDCl_3$) δ 9.31 (d, J = 8.0 Hz, 1H), 8.80 (d, J = 8.3 Hz, 1H), 8.77 – 8.66 (m, 2H), 8.64 (d, J = 8.0 Hz, 1H), 8.54 (d, J = 8.1 Hz, 1H), 8.53 – 8.45 (m, 1H), 7.92 (d, J = 7.9 Hz, 1H), 7.55 – 7.48 (m, 1H), 7.25 – 7.20 (m, 1H), 5.32 – 5.18 (m, 2H), 2.37 – 2.22 (m, 4H), 1.97 – 1.84 (m, 4H), 1.37 – 1.21 (m, 32H), 0.88 – 0.79 (m, 12H). ^{13}C NMR (126 MHz, $CDCl_3$) δ 164.7, 164.4, 164.1, 163.6, 148.0, 147.3, 144.4, 135.5, 134.5, 133.7, 133.6, 132.2, 132.0, 131.6, 131.3, 130.9, 130.5, 130.3, 129.8, 129.6, 129.24, 129.15, 127.33, 127.28, 125.9, 124.0, 123.6, 123.2, 123.1, 122.4, 121.8, 115.6, 114.9, 55.1, 55.0, 32.6, 32.5, 31.9, 29.4, 29.3, 27.14, 27.11, 22.71, 22.68, 14.1. HRMS (APCI) calcd for $C_{56}H_{65}N_2O_4S^+$ $[M + H]^+$: 861.4660; Found: 861.4652.

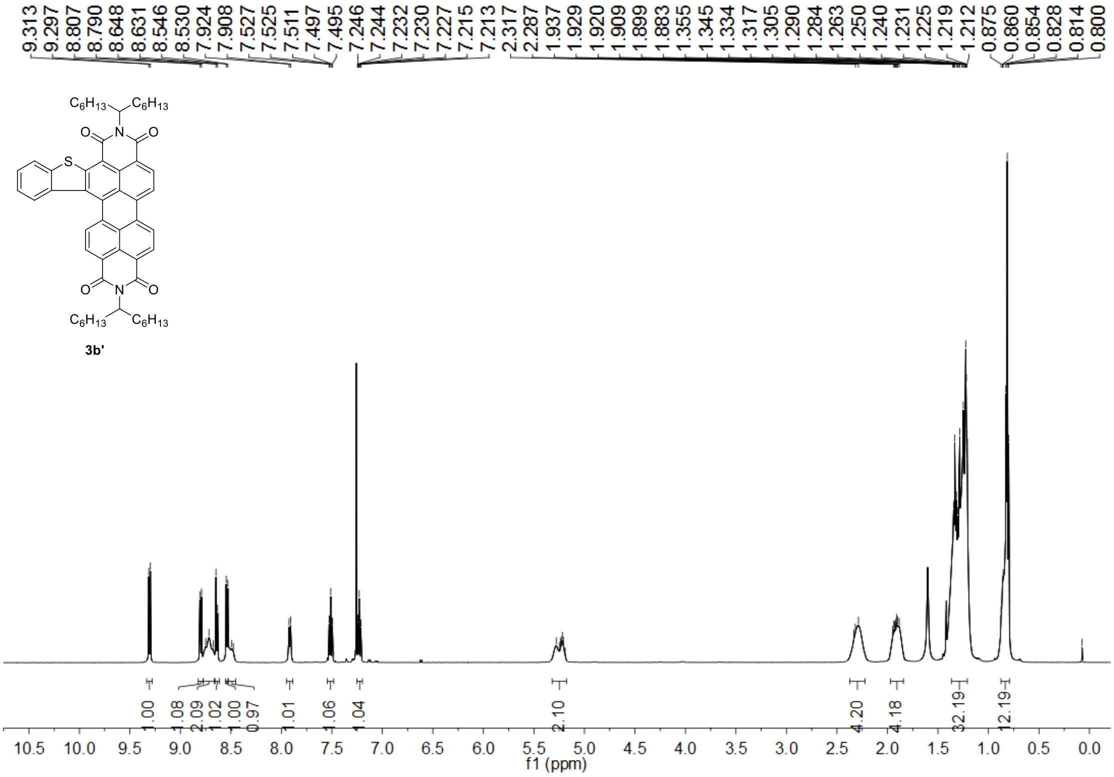

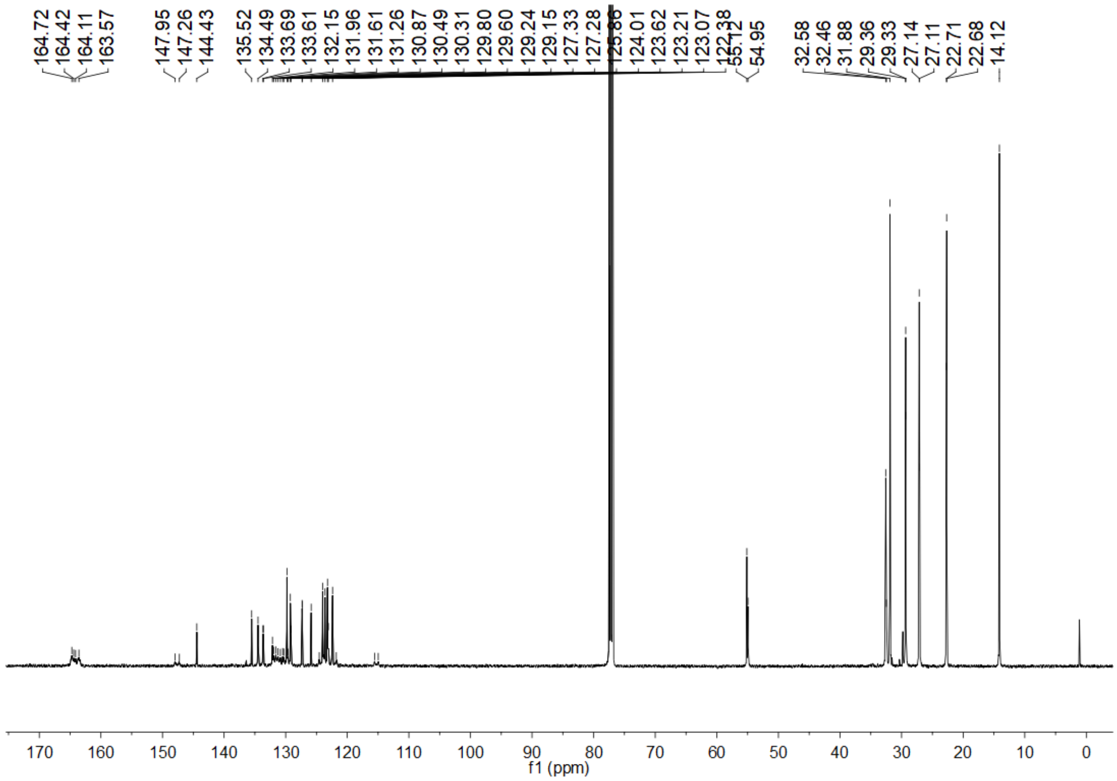

Supplementary Note 2

Synthesis of 2a. To a 15-mL Schlenk tube, PDI **1** (0.1 mmol, 126 mg), KO^{t}Bu (0.6 mmol, 67.3 mg) and *o*-bromoaniline (1 mL) were added under argon. Then, the tube was sealed and the mixture was stirred at 120 ℃ for 24 hours. After cooling down to ambient temperature, 10 mL of methanol was added and precipitation was collected by filtration. Then, the precipitation was purified by neutral alumina column chromatography using DCM/hexane as eluent to afford the red product **2a**. (116.4 mg, 81%)

2a: ^{1}H NMR (500 MHz, $CDCl_3$) δ 11.95 (d, J = 11.0 Hz, 4H), 7.69 (d, J = 7.3 Hz, 4H), 7.55 (d, J = 22.9 Hz, 4H), 7.45 (d, J = 7.6 Hz, 4H), 7.23 (t, J = 7.5 Hz, 4H), 7.11 (t, J = 7.5 Hz, 4H), 5.37 – 5.29 (m, 2H), 2.35 – 2.25 (m, 4H), 1.94 – 1.83 (m, 4H), 1.37 – 1.23 (m, 32H), 0.83 (t, J = 6.8 Hz, 12H). ^{13}C NMR (126 MHz, $CDCl_3$) δ 167.0, 166.6, 150.7, 138.2, 135.0, 134.9, 134.3, 134.0, 128.5, 126.8, 126.7, 126.4, 126.0, 120.5, 120.3, 113.6, 107.3, 101.7, 101.3, 54.1, 32.5, 31.9, 29.4, 27.2, 22.8, 14.2. HRMS (ESI) calcd for $C_{74}H_{78}Br_4N_6O_4Na^+$ [M + Na]$^+$: 1457.2669; Found: 1457.2676.

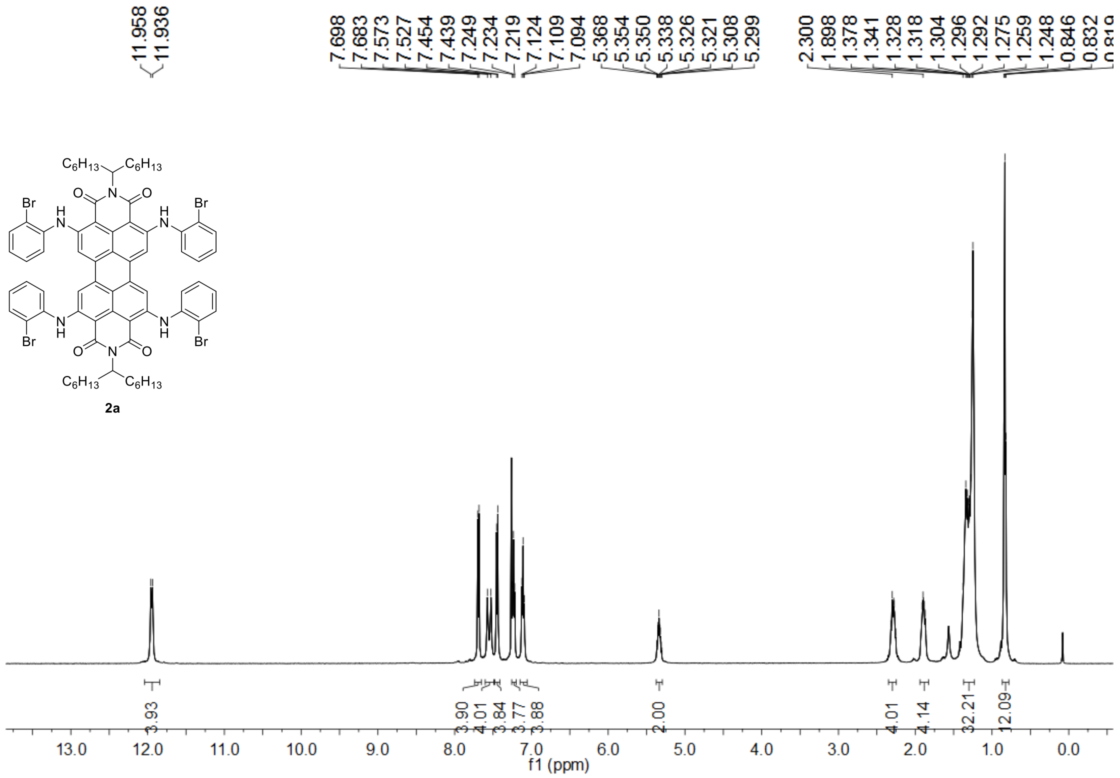

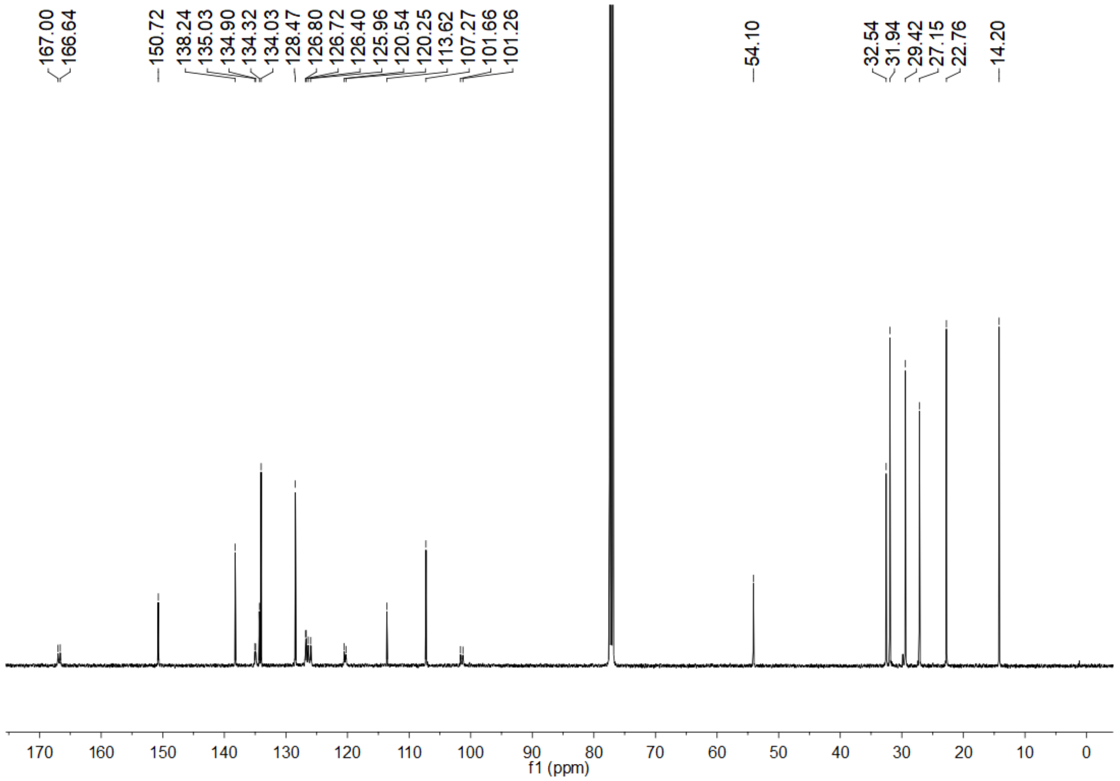

Synthesis of 2b. PDI **1** (0.5 mmol, 629 mg) was dissolved in a mixture of toluene (150 mL) and water (50 mL) bubbled by argon for 30 minutes, CuI (40 mol %, 0.2 mmol, 38 mg,), L-proline (80 mol%, 0.4 mmol, 46 mg), Cs_2CO_3 (4 mmol, 1303.3 mg), CTAB (0.5 mmol, 182.2 mg) were added into the solution under argon. Then *o*-aminophenthiol (4 mmol, 428 uL) was added and the reaction mixture was refluxed overnight at 120 ℃. After the reaction cooled down to room temperature, the

product was extracted with DCM, then, the organic phase was washed with brine and dried with Na_2SO_4. After removal of the solvent, the resident solid was purified by silica gel column chromatography using DCM/hexane as eluent to afford the red product **2b** (606 mg, 97%).

2b: ^{1}H NMR (500 MHz, $CDCl_3$) δ 7.45 – 7.38 (m, 8H), 7.34 (t, J = 7.6 Hz, 4H), 6.84 (t, J = 7.4 Hz, 4H), 6.79 (d, J = 7.9 Hz, 4H), 5.30 – 5.22 (m, 2H), 4.00 (s, 8H), 2.34 – 2.21 (m, 4H), 2.02 – 1.88 (m, 4H), 1.37 – 1.20 (m, 32H), 0.83 (t, J = 6.8 Hz, 12H). ^{13}C NMR (126 MHz, $CDCl_3$) δ 164.5, 163.8, 149.1, 137.1, 133.2, 131.7, 131.2, 122.2, 121.6, 119.5, 117.4, 115.8, 114.6, 55.1, 32.4, 31.9, 29.3, 27.2, 22.7, 14.2. HRMS (ESI) calcd for $C_{74}H_{70}N_2O_4S_4Na^+$ $[M + Na]^+$: 1269.5173; Found: 1269.5196.

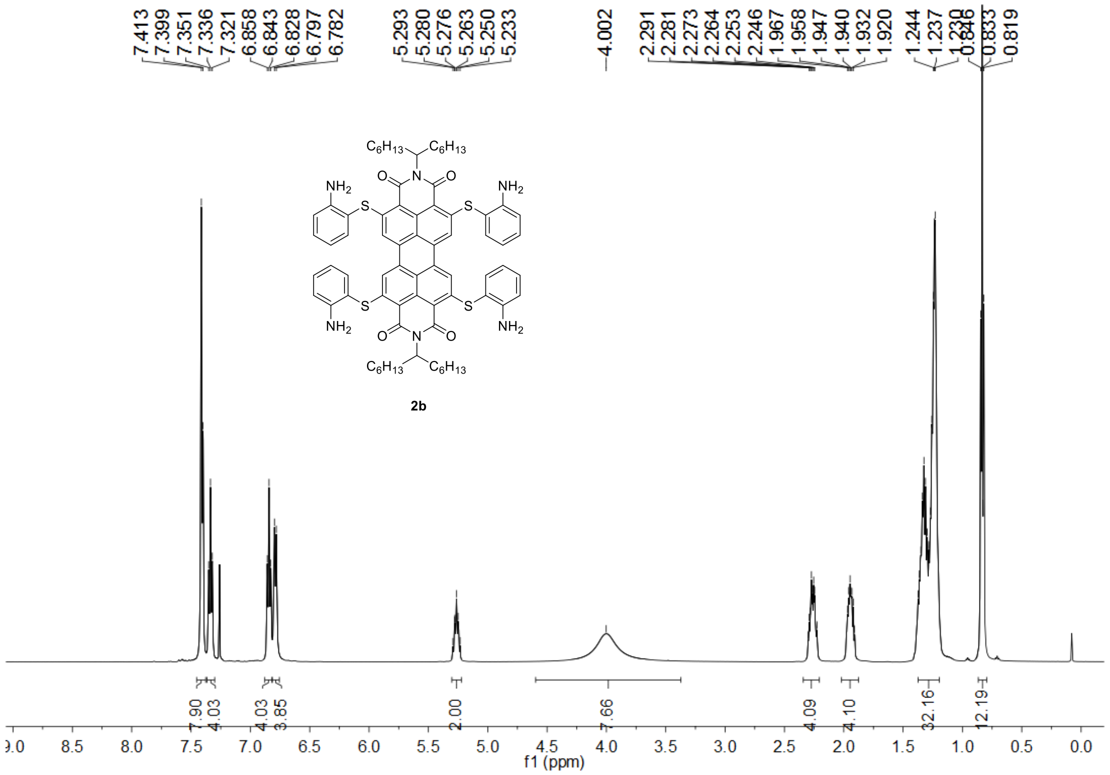

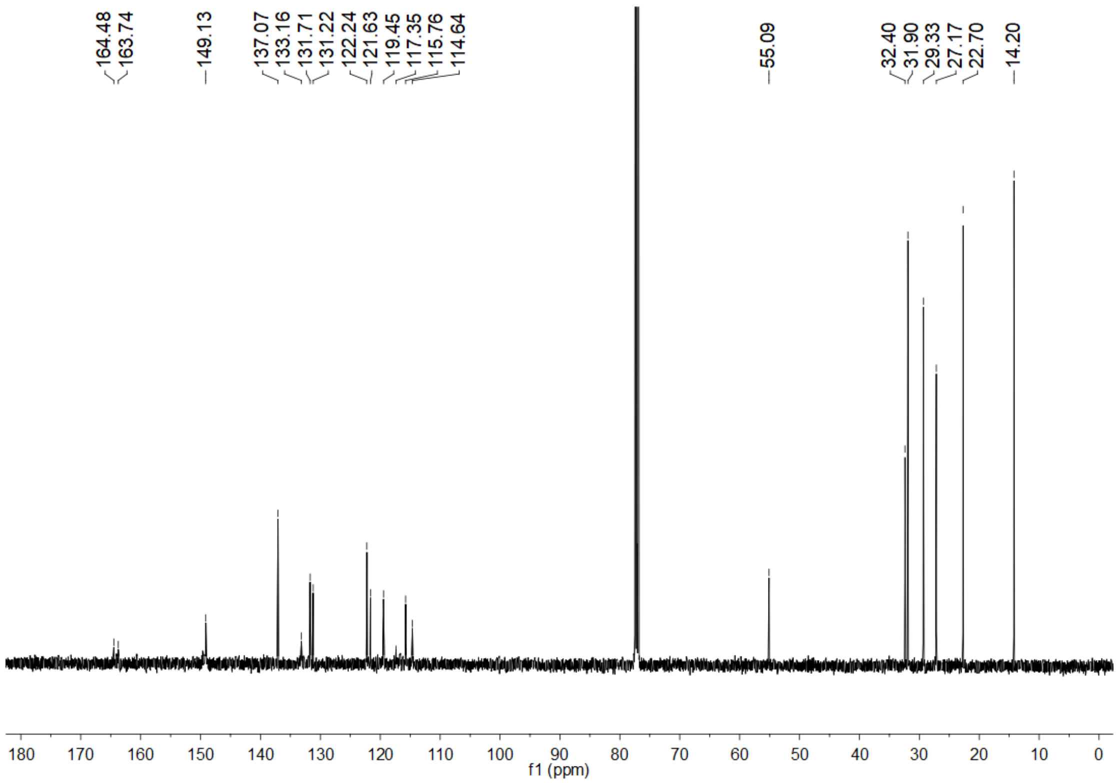

Synthesis of 3a. To a 15-mL Schlenk tube, **2a** (0.05 mmol, 71.7 mg), $Pd(OAc)_2$ (10 mol%, 0.005 mmol, 1.1 mg), P ^{t}Bu$_3$ HBF_4 (20 mol%, 0.01 mmol, 2.9 mg), Cs_2CO_3 (0.4 mmol, 130.3 mg) and dry DMF (2 mL) were added under argon, the mixture was stirred at 150 ℃ for 24 hours. After the reaction cooled down to room temperature, 10 mL of water was added, the mixture was extracted by DCM, then, the organic phase was washed with brine and dried with Na_2SO_4. After removal of the solvent, the resident solid was purified by silica gel column chromatography using DCM/hexane as eluent to afford the mazarine product **3a**. (28.1 mg, 51%)

3a: ^{1}H NMR (500 MHz, $CDCl_3$) δ 11.25 (d, J = 1.7 Hz, 4H), 7.49 (t, J = 7.4 Hz, 4H), 7.29 (t, J = 6.9 Hz, 4H), 6.76 (d, J = 8.1 Hz, 4H), 6.52 (t, J = 7.5 Hz, 4H), 5.60 – 5.53 (m, 2H), 2.59 – 2.48 (m, 4H), 2.15 – 2.03 (m, 4H), 1.56 – 1.40 (m, 16H), 1.31 – 1.25 (m, 16H), 0.85 (t, J = 7.0 Hz, 12H). ^{13}C NMR (126 MHz, $CDCl_3$) δ 167.1, 166.6, 143.7, 143.5, 142.2, 142.1, 129.6, 129.5, 128.6, 125.7, 123.3, 122.9, 122.2, 122.1, 119.8, 119.2, 111.1, 101.5, 100.7, 54.3, 33.1, 33.0, 32.01, 31.97, 29.6, 27.5, 27.4, 22.8, 14.2. HRMS (ESI) calcd for $C_{74}H_{74}N_6O_4Na^+$ [M + Na]$^+$: 1133.5664; Found: 1133.5657.

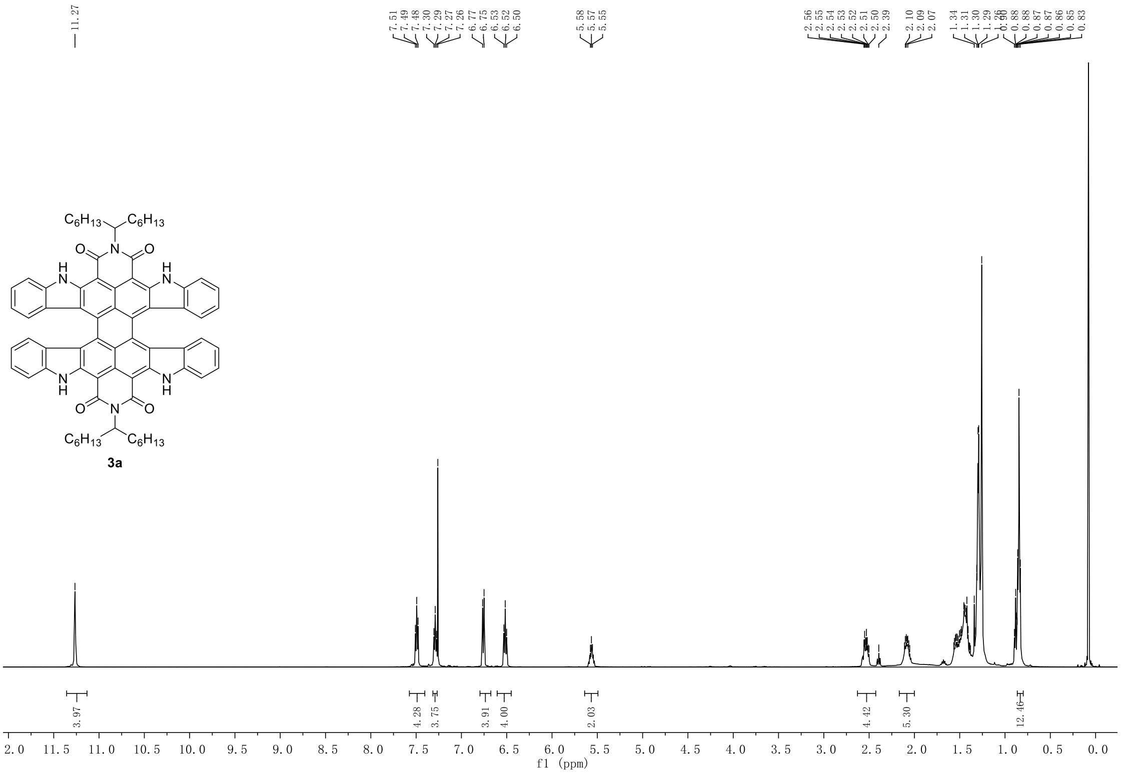

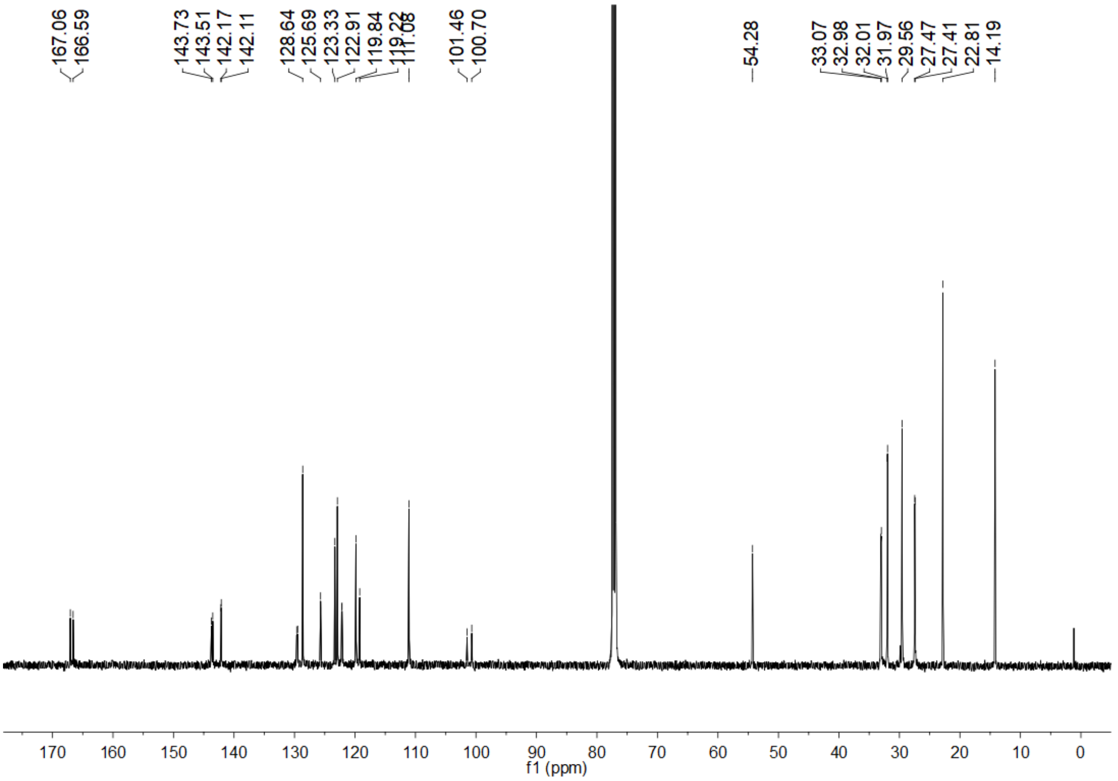

Synthesis of 3b. Dissolve **2b** (0.05 mmol, 62.4 mg) in a mixture of DCM (4 mL) and AcOH (4 mL), isoamyl nitrite (0.6 mmol, 80.6 uL) was injected into the solution while cooling to 0 ℃ and, the diazonium salt was continued to stir in the ice water bath for 1 hours. Then hydroquinone (0.3 mmol, 33 mg) was added to the reaction, the mixture was warmed up to room temperature and continued to stir for additional 30 minutes. The organic solvent was removed by rotary evaporation, the violet product **3b** was obtained by silica gel column chromatography using DCM/hexane as eluent. (41.3

mg, 70%)

3b: ^{1}H NMR (500 MHz, $CDCl_3$) δ 7.78 – 7.65 (m, 4H), 7.19 (t, *J* = 7.4 Hz, 4H), 7.13 (d, *J* = 8.3 Hz, 4H), 6.66 (t, *J* = 7.5 Hz, 4H), 5.58 – 5.46 (m, 2H), 2.55 – 2.41 (m, 4H), 2.20 – 2.07 (m, 4H), 1.56 – 1.28 (m, 32H), 0.98 – 0.82 (m, 12H). ^{13}C NMR (101 MHz, $CDCl_3$) δ 165.0, 164.3, 146.4, 145.6, 143.4, 136.2, 133.43, 133.36, 130.0, 128.1, 128.0, 126.1, 125.2, 124.1, 122.7, 122.2, 114.7, 114.0, 55.6, 32.8, 32.6, 32.0, 31.9, 29.5, 29.4, 27.4, 22.78, 22.76, 14.22, 14.19. HRMS (ESI) calcd for $C_{74}H_{70}N_2O_4S_4Na^+$ $[M + Na]^+$: 1201.4111; Found: 1201.4135.

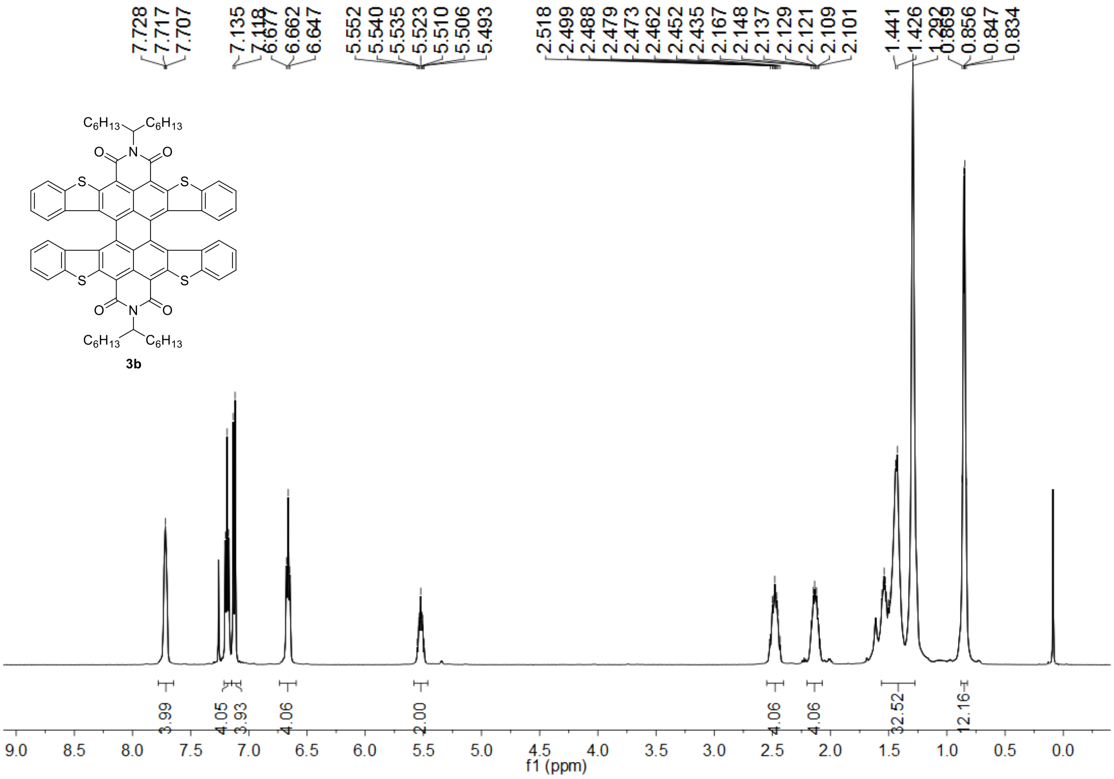

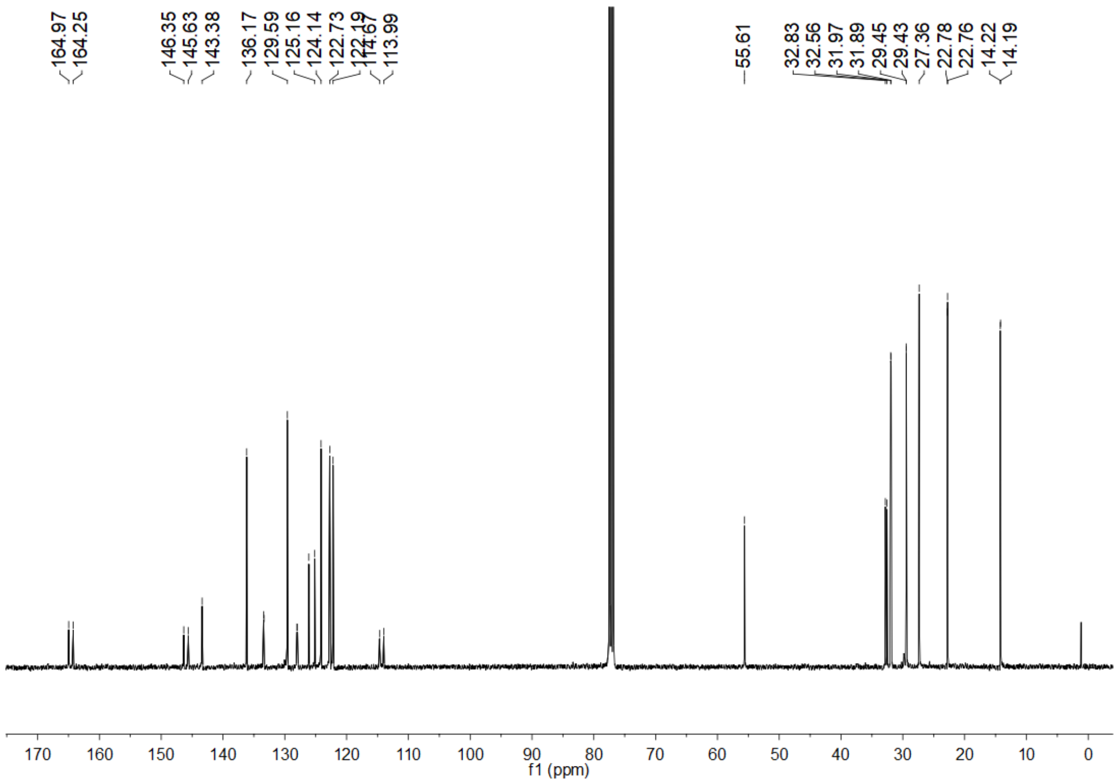

Supplementary Note 3: General Information and Methods

All commercial reagents were used without further purification unless otherwise indicated. All oxygen sensitive reactions were performed under argon atmosphere using standard Schlenk method. ^{1}H and ^{13}C NMR spectra were recorded at 400 or 500 MHz in $CDCl_3$ as solvent. High-resolution mass spectra (HRMS) were obtained on a FT-MS instrument using ESI or APCI technique. Absorption spectra were recorded on a Thermo Scientific Evolution 300 UV/Vis spectrophotometer. CD spectra of solutions were recorded with a JASCO J-1500 spectropolarimeter. Absorption and CD spectra of thin films were recorded with a JASCO V-770 and a JASCO-815 150-L with an equipment for solid samples, respectively. For transmission spectroscopy, we thermally deposited thin films on transparent quartz plates. Cyclic voltammetry (CV) measurements were performed on a Bio-Logic-Science Instrument EC-LAB SP-200. The g_{abs} values are typically defined by the following equation:

$$g_{\text{abs}} = \frac{\theta}{33000 * Abs} \qquad \text{Supplementary Equation 1}$$

Optimizations of minima and transition state were performed with the ωB97Xd[2] functional in order to include dispersion corrections in combination with the 6-31+G(d) basis set. The same combination of functional and basis set was used to calculate the circular dichroism spectra, the first 20 states were calculated for the [7]-heterohelicenes molecules and the first 40 states for the perylene diimide double-heterohelicenes. A gaussian broadening of 0.2 eV was used. Alkyl chains were substituted by a methyl groups, as alkyl chains do not contribute to the CD signals.

OFET fabrication: OFETs based on **3a** and **3b** were fabricated using heavily doped silicon wafers

covered with a 300-nm-thick SiO_2 layer (C_i=11.5 nF cm^{-2}). Wafers were cleaned with a piranha solution for 30 min, followed by UV-ozone treatment. The wafer surface was treated with an OTS self-assembled monolayer. The OTS solution (3 mM in trichloroethylene) was spin-coated onto the wafers at 1500 rpm for 30 s, and the samples then kept overnight in a vacuum desiccator with a separate vial containing NH_4OH. The wafers were then washed with toluene, acetone, and isopropyl alcohol and dried under nitrogen. To fabricate thin films, PDI derivatives were deposited (40 nm) onto OTS-treated wafers in a thermal evaporator at different substrate temperatures. The chamber was under high vacuum ($< 5.0 \times 10^{-6}$ torr), and the deposition rate was maintained at 0.2 Å s^{-1}. Gold electrodes (40 nm) were thermally evaporated and patterned using shadow masks. The source/drain patterns had a channel length (L) of 50 μm and a channel width (W) of 1000 μm (W/L = 20).

Electrical Measurements: Current–voltage characteristics of OFETs were measured inside a vacuum chamber, using the Keithley 4200-SCS semiconductor parametric analyser. To investigate photocurrent of phototransistor devices, laser instrument (CNI laser, maximum power of 5 mW) was used to generate monochromatic light. For testing the spectral photoresponse, monochromatic light was produced using an 300W Xenon lamp and Oriel Cornerstone 130 monochromator with dual gratings. 280 μm slits were used for bandwidth of 3.7 nm. The CPL illumination was generated through a linear polarizer and a quarter-wave plate (Thorlabs). To confirm the quality of circular polarization of the light, we tested the intensity independence of the light passing through the quartz as the degree between the transmission axis of the linear polarizer and the fast axis of the quarter-wave plate changed. Also, we rotated the degree to avoid elliptical polarization by confirming that the intensity of light splits into two orthogonal linear polarization state using beam splitter (Thorlabs). All corrections were conducted by placing the Si photodetector in the same position with the samples.

Estimation of Optoelectrical Properties: In order to investigate photosensitivity for OFETs, photoresponsivity (R) and photocurrent/dark-current ratio (P) were calculated from transfer characteristics coupled with light irradiation. The R and P values are typically defined by the following equations:

$$R = \frac{I_{\mathrm{ph}}}{P_{\mathrm{inc}}} = \frac{I_{\mathrm{light}} - I_{\mathrm{dark}}}{P_{\mathrm{inc}}} \qquad \text{Supplementary Equation 2}$$

$$P = \frac{I_{\mathrm{light}} - I_{\mathrm{dark}}}{I_{\mathrm{dark}}} \qquad \text{Supplementary Equation 3}$$

where I_{ph} is the photocurrent, P_{inc} the incident illumination power on the channel of the device, I_{light} the drain current under illumination, and I_{dark} the drain current in the dark, respectively. In addition, the external quantum efficiency (EQE) (η) of OPTs was calculated which can be defined as the ratio of number of photogenerated carriers that practically enhances the drain current to the number of photons incident onto the OPT channel area, using the following equation:

$$\eta = \frac{(I_{light} - I_{dark})hc}{eP_{int}A\lambda_{peak}} \qquad \text{Supplementary Equation 4}$$

where h is the plank constant, c the speed of light, e the fundamental unit of charge, P_{int} the incident power density, A the area of the transistor channel, and λ_{peak} the peak wavelength of the incident

light, respectively.

Detectivity usually describes the smallest detectable signal, which allows comparisons of phototransistor devices with different configurations and areas. D^* was evaluated within this study using the following Supplementary Equations (5) and (6):

$$D^* = \frac{\sqrt{A}}{NEP}$$ Supplementary Equation 5

$$NEP = \frac{\sqrt{\overline{I_n^2}}}{R\sqrt{\Delta f}}$$ Supplementary Equation 6

In these equations, A is the phototransistor active area, NEP the noise equivalent power, $\overline{I_n^2}$ the measured noise current, and Δf the bandwidth. If the major limit to detectivity is shot noise from the drain current under dark conditions, D^* can be simplified as:

$$D^* = \frac{R}{\sqrt{(2e \cdot I_{dark}/A)}}$$ Supplementary Equation 7

Supplementary Note 4: The cartesian coordinates of all the optimized structures.

3a-*P*:

N	-0.001807	5.668885	-0.005829
C	1.169514	5.019484	-0.406104
C	-1.165389	5.015542	0.399841
C	1.156388	3.562245	-0.377087
C	-1.156734	3.559894	0.376393
C	0.000196	2.845257	-0.000086
C	2.265093	2.827389	-0.782370
C	-2.265051	2.826885	0.783510
C	-0.000011	1.423181	0.000741
C	1.221936	0.708928	-0.191289
C	-2.313764	1.394262	0.721037
C	-1.222028	0.709072	0.193661
C	2.313226	1.394283	-0.718782
C	-1.221936	-0.708928	-0.191289
C	0.000011	-1.423181	0.000741
C	1.222028	-0.709072	0.193661
O	-2.142567	5.673231	0.759871
C	-0.037118	7.129174	0.001181
O	2.154979	5.663193	-0.767937
H	0.921685	7.489999	-0.364895
H	-0.217701	7.490519	1.016011
H	-0.846925	7.480006	-0.641891
C	-2.313226	-1.394283	-0.718782
C	2.313764	-1.394262	0.721037

C	-0.000196	-2.845257	-0.000086
C	-2.265093	-2.827389	-0.782370
C	-1.156388	-3.562245	-0.377087
C	2.265051	-2.826885	0.783510
C	1.156734	-3.559894	0.376393
C	1.165389	-5.015542	0.399841
C	-1.169514	-5.019484	-0.406104
N	0.001807	-5.668885	-0.005829
O	-2.154979	-5.663193	-0.767937
O	2.142567	-5.673231	0.759871
C	0.037118	-7.129174	0.001181
H	-0.921685	-7.489999	-0.364895
H	0.217701	-7.490519	1.016011
H	0.846925	-7.480006	-0.641891
C	3.599280	-1.021062	1.299264
N	3.446388	-3.294089	1.267998
C	4.262497	-2.227140	1.611416
H	3.617184	-4.283976	1.391428
C	4.209228	0.189842	1.642988
C	5.463188	0.172610	2.238186
C	5.524528	-2.257136	2.197968
C	6.119492	-1.038521	2.499835
H	6.018162	-3.198384	2.420170
H	7.103869	-1.025929	2.958496
H	3.713728	1.135109	1.454197
H	5.943547	1.110471	2.498942
C	3.598357	1.019564	-1.296892
N	3.447093	3.292457	-1.266911
C	4.262502	2.224786	-1.609587
H	3.618225	4.282246	-1.390558
C	4.207034	-0.191975	-1.640632
C	5.524528	2.253627	-2.196172
C	5.460970	-0.176014	-2.235949
H	3.710647	-1.136775	-1.451953
C	6.118442	1.034419	-2.497795
H	6.018898	3.194404	-2.418725
H	7.102784	1.020798	-2.956500
H	5.940356	-1.114389	-2.496662

C	-3.599280	1.021062	1.299264
C	-4.262497	2.227140	1.611416
N	-3.446388	3.294089	1.267998
H	-3.617184	4.283976	1.391428
C	-5.524528	2.257136	2.197968
C	-4.209228	-0.189842	1.642988
C	-5.463188	-0.172610	2.238186
H	-3.713728	-1.135109	1.454197
C	-6.119492	1.038521	2.499835
H	-6.018162	3.198384	2.420170
H	-7.103869	1.025929	2.958496
H	-5.943547	-1.110471	2.498942
N	-3.447093	-3.292457	-1.266911
C	-3.598357	-1.019564	-1.296892
C	-4.262502	-2.224786	-1.609587
H	-3.618225	-4.282246	-1.390558
C	-5.524528	-2.253627	-2.196172
C	-4.207034	0.191975	-1.640632
C	-6.118442	-1.034419	-2.497795
H	-6.018898	-3.194404	-2.418725
C	-5.460970	0.176014	-2.235949
H	-3.710647	1.136775	-1.451953
H	-7.102784	-1.020798	-2.956500
H	-5.940356	1.114389	-2.496662

3a-*M*:

N	0.002566	5.668887	-0.005690
C	-1.168896	5.019652	-0.405828
C	1.166092	5.015390	0.399948
C	-1.155972	3.562439	-0.376838
C	1.157304	3.559745	0.376320
C	0.000183	2.845279	0.000016
C	-2.264781	2.827707	-0.782101
C	2.265563	2.826581	0.783310
C	0.000183	1.423223	0.000827
C	-1.221836	0.709083	-0.191144
C	2.313939	1.393953	0.721038
C	1.222101	0.708923	0.193740

C	-2.313038	1.394596	-0.718617
C	1.221836	-0.709083	-0.191144
C	-0.000183	-1.423223	0.000827
C	-1.222101	-0.708923	0.193740
O	2.143323	5.672948	0.760036
C	0.038081	7.129165	0.001387
O	-2.154311	5.663507	-0.767587
H	-0.920539	7.490123	-0.365047
H	0.218360	7.490460	1.016288
H	0.848176	7.479835	-0.641397
C	2.313038	-1.394596	-0.718617
C	-2.313939	-1.393953	0.721038
C	-0.000183	-2.845279	0.000016
C	2.264781	-2.827707	-0.782101
C	1.155972	-3.562439	-0.376838
C	-2.265563	-2.826581	0.783310
C	-1.157304	-3.559745	0.376320
C	-1.166092	-5.015390	0.399948
C	1.168896	-5.019652	-0.405828
N	-0.002566	-5.668887	-0.005690
O	2.154311	-5.663507	-0.767587
O	-2.143323	-5.672948	0.760036
C	-0.038081	-7.129165	0.001387
H	0.920539	-7.490123	-0.365047
H	-0.218360	-7.490460	1.016288
H	-0.848176	-7.479835	-0.641397
C	-3.599406	-1.020500	1.299150
N	-3.447215	-3.293553	1.267294
C	-4.263043	-2.226436	1.610913
H	-3.618017	-4.283374	1.391267
C	-4.208994	0.190558	1.642935
C	-5.463062	0.173614	2.237913
C	-5.525221	-2.256126	2.197150
C	-6.119815	-1.037363	2.499190
H	-6.019275	-3.197234	2.419019
H	-7.104295	-1.024562	2.957624
H	-3.713123	1.135690	1.454349
H	-5.943156	1.111574	2.498821

C	-3.598204	1.020003	-1.296707
N	-3.446800	3.292873	-1.266538
C	-4.262268	2.225281	-1.609353
H	-3.617742	4.282626	-1.390654
C	-4.206871	-0.191491	-1.640606
C	-5.524266	2.254171	-2.195988
C	-5.460813	-0.175482	-2.235896
H	-3.710508	-1.136303	-1.451947
C	-6.118224	1.034993	-2.497706
H	-6.018601	3.194954	-2.418616
H	-7.102570	1.021440	-2.956409
H	-5.940240	-1.113823	-2.496658
C	3.599406	1.020500	1.299150
C	4.263043	2.226436	1.610913
N	3.447215	3.293553	1.267294
H	3.618017	4.283374	1.391267
C	5.525221	2.256126	2.197150
C	4.208994	-0.190558	1.642935
C	5.463062	-0.173614	2.237913
H	3.713123	-1.135690	1.454349
C	6.119815	1.037363	2.499190
H	6.019275	3.197234	2.419019
H	7.104295	1.024562	2.957624
H	5.943156	-1.111574	2.498821
N	3.446800	-3.292873	-1.266538
C	3.598204	-1.020003	-1.296707
C	4.262268	-2.225281	-1.609353
H	3.617742	-4.282626	-1.390654
C	5.524266	-2.254171	-2.195988
C	4.206871	0.191491	-1.640606
C	6.118224	-1.034993	-2.497706
H	6.018601	-3.194954	-2.418616
C	5.460813	0.175482	-2.235896
H	3.710508	1.136303	-1.451947
H	7.102570	-1.021440	-2.956409
H	5.940240	1.113823	-2.496658

3a-*meso*:

N	-5.657020	-0.635412	0.000000
C	-5.022649	-0.466741	1.230464
C	-5.022649	-0.466741	-1.230464
C	-3.576007	-0.292359	1.211470
C	-3.576007	-0.292359	-1.211470
C	-2.852056	-0.261978	0.000000
C	-2.892524	0.071616	2.364284
C	-2.892524	0.071616	-2.364284
C	-1.431178	-0.134821	0.000000
C	-0.732172	0.029068	1.240445
C	-1.487594	0.348206	-2.373358
C	-0.732172	0.029068	-1.240445
C	-1.487594	0.348206	2.373358
C	0.732147	-0.029193	-1.240437
C	1.431147	0.134717	-0.000000
C	0.732147	-0.029193	1.240437
O	-5.680115	-0.463110	-2.271827
C	-7.104366	-0.830835	0.000000
O	-5.680115	-0.463110	2.271827
H	-7.378485	-1.382004	0.898123
H	-7.621047	0.133547	0.000000
H	-7.378485	-1.382004	-0.898123
C	1.487575	-0.348322	-2.373348
C	1.487575	-0.348322	2.373348
C	2.852033	0.261929	-0.000000
C	2.892463	-0.071591	-2.364309
C	3.575957	0.292369	-1.211492
C	2.892463	-0.071591	2.364309
C	3.575957	0.292369	1.211492
C	5.022630	0.466766	1.230458
C	5.022630	0.466766	-1.230458
N	5.656945	0.635755	-0.000000
O	5.680202	0.462685	-2.271737
O	5.680202	0.462685	2.271737
C	7.104318	0.830831	-0.000000
H	7.378564	1.381934	-0.898148
H	7.378564	1.381934	0.898148
H	7.620849	-0.133639	-0.000000

C	1.227868	-0.970258	3.669312
N	3.410100	-0.319055	3.597709
C	2.439661	-0.913175	4.389652
H	4.402761	-0.238305	3.779289
C	0.139669	-1.633123	4.248580
C	0.266229	-2.164774	5.524056
C	2.574858	-1.426156	5.676602
C	1.467147	-2.047136	6.238444
H	3.517673	-1.356915	6.210798
H	1.539325	-2.462826	7.239240
H	-0.796436	-1.739240	3.712471
H	-0.579716	-2.673395	5.975777
C	-1.227896	0.970304	3.669244
N	-3.410173	0.319357	3.597614
C	-2.439682	0.913285	4.389599
H	-4.402695	0.237776	3.779542
C	-0.139640	1.633102	4.248479
C	-2.574839	1.426335	5.676532
C	-0.266137	2.164744	5.523965
H	0.796475	1.739109	3.712363
C	-1.467065	2.047216	6.238359
H	-3.517660	1.357213	6.210732
H	-1.539205	2.462920	7.239152
H	0.579870	2.673250	5.975699
C	-1.227896	0.970304	-3.669244
C	-2.439682	0.913285	-4.389599
N	-3.410173	0.319357	-3.597614
H	-4.402695	0.237776	-3.779542
C	-2.574839	1.426335	-5.676532
C	-0.139640	1.633102	-4.248479
C	-0.266137	2.164744	-5.523965
H	0.796475	1.739109	-3.712363
C	-1.467065	2.047216	-6.238359
H	-3.517660	1.357213	-6.210732
H	-1.539205	2.462920	-7.239152
H	0.579870	2.673250	-5.975699
N	3.410100	-0.319055	-3.597709
C	1.227868	-0.970258	-3.669312

C	2.439661	-0.913175	-4.389652
H	4.402761	-0.238305	-3.779289
C	2.574858	-1.426156	-5.676602
C	0.139669	-1.633123	-4.248580
C	1.467147	-2.047136	-6.238444
H	3.517673	-1.356915	-6.210798
C	0.266229	-2.164774	-5.524056
H	-0.796436	-1.739240	-3.712471
H	1.539325	-2.462826	-7.239240
H	-0.579716	-2.673395	-5.975777

3a-*P*-TS:

C	-2.470941	2.871684	-0.302695
C	-2.541506	1.579691	0.325828
C	-1.367577	0.784306	0.348936
C	-0.110340	1.505569	0.172596
C	1.161426	0.844203	0.231397
C	2.339794	1.529729	-0.094667
C	2.297351	2.954928	-0.176049
C	-2.120783	-2.694163	-0.991780
C	-2.351439	-1.650392	-0.031860
C	-1.301943	-0.713973	0.178420
C	0.004251	-1.288287	-0.009397
C	1.167422	-0.593999	0.428792
C	2.242741	-1.347437	0.889466
C	2.359874	-2.708088	0.466367
C	4.174954	-2.268761	1.713308
C	3.408670	-1.084611	1.714250
C	3.846180	0.013128	2.458919
C	5.373935	-2.376443	2.411732
C	5.038719	-0.084437	3.161747
C	5.796354	-1.265172	3.131918
H	5.954920	-3.293728	2.396092
H	6.730924	-1.314489	3.683074
H	5.394995	0.765731	3.735058
H	3.272024	0.934248	2.473154
C	3.718684	1.175804	-0.457654
C	4.424277	2.394628	-0.564385

C	5.772459	2.467633	-0.902027
H	6.283025	3.424477	-0.954843
C	4.393393	0.003764	-0.825106
H	3.882424	-0.948928	-0.859309
C	6.424725	1.279418	-1.201459
C	5.731669	0.062133	-1.188535
H	6.244995	-0.853218	-1.466256
H	7.476252	1.298874	-1.472598
C	-4.249094	-2.929734	-0.393078
C	-3.672618	-2.000431	0.502822
C	-4.205457	-1.971123	1.795259
C	-5.481677	-3.534289	-0.166704
C	-6.076880	-3.330232	1.072737
C	-5.408355	-2.608388	2.069830
H	-5.914708	-4.209043	-0.898864
H	-7.026443	-3.807759	1.295594
H	-5.817288	-2.572318	3.075053
H	-3.639330	-1.525449	2.603672
C	-4.644037	2.539155	0.059425
C	-3.961257	1.478053	0.695623
C	-4.644734	0.837833	1.726455
H	-4.103151	0.212605	2.411095
C	-6.005361	2.778865	0.220112
H	-6.495348	3.586670	-0.314886
C	-5.996709	1.072616	1.934164
C	-6.687452	2.003181	1.149009
H	-6.513030	0.545681	2.730576
H	-7.747939	2.169787	1.313156
C	-0.855530	-3.214861	-1.230428
C	0.193749	-2.600948	-0.527809
C	1.409909	-3.295080	-0.360924
C	-1.291279	3.565858	-0.489846
C	-0.100621	2.901901	-0.150525
C	1.098249	3.647198	-0.231139
C	1.110338	5.068935	-0.561863
O	2.147799	5.732788	-0.583986
C	-1.313020	4.958280	-0.923253
O	-2.340485	5.542703	-1.271948

C	1.610303	-4.612727	-0.940963
O	2.635276	-5.268920	-0.755065
C	-0.671227	-4.503879	-1.884684
O	-1.564502	-5.071451	-2.513171
N	3.552397	3.447228	-0.340236
N	-3.311772	-3.257273	-1.363036
N	-3.718700	3.333884	-0.584081
N	3.531416	-3.219773	0.931001
H	3.836679	-4.156398	0.700886
H	3.707967	4.432016	-0.517420
H	-3.849039	4.278909	-0.927617
H	-3.304636	-4.124287	-1.889979
N	-0.110277	5.651687	-0.896886
N	0.578499	-5.110776	-1.744266
C	-0.155716	7.058508	-1.276616
C	0.760077	-6.410020	-2.379316
H	-0.760948	7.618846	-0.559978
H	0.148266	-7.167584	-1.881529
H	0.449634	-6.342012	-3.422655
H	1.812699	-6.678254	-2.304978
H	0.865539	7.433341	-1.283339
H	-0.607818	7.159307	-2.265339

3a-*M*-TS:

C	-2.301886	2.951470	-0.183774
C	-2.341546	1.526435	-0.100840
C	-1.163100	0.843798	0.230552
C	0.107701	1.507146	0.174648
C	1.365907	0.787068	0.349954
C	2.538803	1.583728	0.327757
C	2.466546	2.877636	-0.296867
C	-2.355626	-2.710708	0.477099
C	-2.241723	-1.347749	0.894231
C	-1.167823	-0.594055	0.430704
C	-0.003944	-1.287264	-0.006931
C	1.301783	-0.711214	0.178122
C	2.352067	-1.646230	-0.035863
C	2.120010	-2.688236	-0.997468

C	4.249473	-2.924259	-0.403455
C	3.673966	-1.997700	0.496007
C	4.208096	-1.972531	1.788039
C	5.482419	-3.529342	-0.180214
C	5.411136	-2.610793	2.059445
C	6.078723	-3.329372	1.059355
H	5.914843	-4.201739	-0.914893
H	7.028438	-3.807714	1.279806
H	5.820905	-2.578216	3.064451
H	3.643033	-1.528974	2.598415
C	3.959211	1.481720	0.694797
C	4.640536	2.544949	0.060745
C	6.002163	2.784239	0.219417
H	6.491004	3.593878	-0.313845
C	4.644888	0.837912	1.721826
H	4.104665	0.210328	2.405333
C	6.686252	2.005324	1.144143
C	5.997326	1.071918	1.927495
H	6.515378	0.542107	2.720862
H	7.747055	2.171519	1.306678
C	-4.169749	-2.271247	1.725460
C	-3.407174	-1.084784	1.719899
C	-3.846556	0.014621	2.461018
C	-5.366873	-2.379928	2.426908
C	-5.791378	-1.267056	3.143351
C	-5.037335	-0.083862	3.166774
H	-5.944780	-3.299217	2.416359
H	-6.724247	-1.317165	3.697292
H	-5.395074	0.767523	3.737360
H	-3.275102	0.937459	2.470773
C	-4.424902	2.385724	-0.585434
C	-3.717468	1.168710	-0.470991
C	-4.386965	-0.006026	-0.839140
H	-3.873599	-0.957742	-0.866203
C	-5.770713	2.454794	-0.933328
H	-6.282961	3.410364	-0.992638
C	-5.722777	0.048411	-1.212145
C	-6.418140	1.264299	-1.234181

H	-6.232325	-0.868803	-1.490683
H	-7.467568	1.280692	-1.513555
C	-1.405654	-3.298754	-0.349742
C	-0.192455	-2.600974	-0.522879
C	0.854976	-3.211617	-1.230415
C	-1.104266	3.646790	-0.229531
C	0.096132	2.904285	-0.145139
C	1.286177	3.571502	-0.480402
C	1.306882	4.964975	-0.911345
O	2.333036	5.546890	-1.267697
C	-1.117402	5.068926	-0.557716
O	-2.156276	5.730081	-0.593824
C	0.668886	-4.498871	-1.887184
O	1.555765	-5.057774	-2.532581
C	-1.604126	-4.620085	-0.922835
O	-2.628295	-5.275340	-0.729355
N	3.713701	3.341352	-0.578372
N	-3.524411	-3.223909	0.946729
N	-3.556894	3.440742	-0.357358
N	3.310832	-3.249117	-1.373025
H	3.304440	-4.113176	-1.904694
H	3.843425	4.286098	-0.922706
H	-3.713252	4.424959	-0.537311
H	-3.827798	-4.161884	0.719561
N	-0.571286	-5.117329	-1.726286
N	0.104757	5.659360	-0.870702
C	-0.740554	-6.418652	-2.367803
C	0.140695	7.071696	-1.240233
H	-0.033298	-7.138844	-1.949191
H	-0.841352	7.494857	-1.040403
H	0.908196	7.579599	-0.654102
H	0.385270	7.175947	-2.300276
H	-1.762056	-6.747895	-2.189068
H	-0.547681	-6.323143	-3.437934

3b-*P*:

N	-0.000767	5.649645	0.000885
C	1.183069	5.004801	-0.361223

C	-1.179110	5.000252	0.361483
C	1.165954	3.534618	-0.373597
C	-1.166934	3.531962	0.375298
C	-0.000122	2.829975	0.000617
C	2.271836	2.816267	-0.803167
C	-2.272897	2.814677	0.804205
C	-0.000296	1.415822	0.000629
C	1.217059	0.705825	-0.211416
C	-2.298686	1.382282	0.780383
C	-1.217635	0.705416	0.212516
C	2.296960	1.383448	-0.780128
C	-1.217059	-0.705825	-0.211416
C	0.000296	-1.415822	0.000629
C	1.217635	-0.705416	0.212516
O	-2.170991	5.648876	0.664549
C	-0.035807	7.111078	0.009621
O	2.182468	5.639863	-0.666658
H	0.947680	7.472565	-0.282590
H	-0.291336	7.467264	1.009731
H	-0.794596	7.465011	-0.691388
C	-2.296960	-1.383448	-0.780128
C	2.298686	-1.382282	0.780383
C	0.000122	-2.829975	0.000617
C	-2.271836	-2.816267	-0.803167
C	-1.165954	-3.534618	-0.373597
C	2.272897	-2.814677	0.804205
C	1.166934	-3.531962	0.375298
C	1.179110	-5.000252	0.361483
C	-1.183069	-5.004801	-0.361223
N	0.000767	-5.649645	0.000885
O	-2.182468	-5.639863	-0.666658
O	2.170991	-5.648876	0.664549
C	0.035807	-7.111078	0.009621
H	-0.947680	-7.472565	-0.282590
H	0.291336	-7.467264	1.009731
H	0.794596	-7.465011	-0.691388
C	3.537627	-0.870857	1.366766
C	4.421972	-1.912924	1.685826

C	3.897418	0.439044	1.715486
C	5.130372	0.686656	2.296974
C	5.668162	-1.671632	2.263480
C	6.022397	-0.361679	2.553955
H	6.340315	-2.492851	2.494445
H	6.988584	-0.153432	3.003736
H	3.216142	1.263704	1.544017
H	5.402224	1.705508	2.555093
C	3.534545	0.870809	-1.368250
C	4.419854	1.911814	-1.687232
C	3.891976	-0.439279	-1.718699
C	5.665210	1.669409	-2.266247
C	5.124058	-0.688074	-2.301524
H	3.209625	-1.263114	-1.547547
C	6.017392	0.359232	-2.558208
H	6.338280	2.489916	-2.497086
H	6.982903	0.150017	-3.008991
H	5.394205	-1.707049	-2.560956
C	-3.537627	0.870857	1.366766
C	-4.421972	1.912924	1.685826
C	-5.668162	1.671632	2.263480
C	-3.897418	-0.439044	1.715486
C	-5.130372	-0.686656	2.296974
H	-3.216142	-1.263704	1.544017
C	-6.022397	0.361679	2.553955
H	-6.340315	2.492851	2.494445
H	-6.988584	0.153432	3.003736
H	-5.402224	-1.705508	2.555093
C	-3.534545	-0.870809	-1.368250
C	-4.419854	-1.911814	-1.687232
C	-5.665210	-1.669409	-2.266247
C	-3.891976	0.439279	-1.718699
C	-6.017392	-0.359232	-2.558208
H	-6.338280	-2.489916	-2.497086
C	-5.124058	0.688074	-2.301524
H	-3.209625	1.263114	-1.547547
H	-6.982903	-0.150017	-3.008991
H	-5.394205	1.707049	-2.560956

S	3.774946	-3.514062	1.350950
S	-3.774946	-3.513300	-1.350517
S	-3.774946	3.514062	1.350950
S	3.774946	3.513300	-1.350517

3b-*M*:

N	0.000852	5.649643	0.000652
C	-1.183049	5.004819	-0.361277
C	1.179192	5.000244	0.361237
C	-1.165953	3.534640	-0.373644
C	1.166970	3.531964	0.375143
C	0.000130	2.829990	0.000515
C	-2.271883	2.816304	-0.803123
C	2.272914	2.814671	0.804071
C	0.000284	1.415832	0.000549
C	-1.217077	0.705832	-0.211437
C	2.298663	1.382286	0.780308
C	1.217623	0.705417	0.212432
C	-2.297007	1.383484	-0.780076
C	1.217077	-0.705832	-0.211437
C	-0.000284	-1.415832	0.000549
C	-1.217623	-0.705417	0.212432
O	2.171112	5.648848	0.664223
C	0.035958	7.111071	0.009284
O	-2.182484	5.639887	-0.666576
H	-0.947456	7.472611	-0.283107
H	0.291331	7.467333	1.009409
H	0.794887	7.464906	-0.691623
C	2.297007	-1.383484	-0.780076
C	-2.298663	-1.382286	0.780308
C	-0.000130	-2.829990	0.000515
C	2.271883	-2.816304	-0.803123
C	1.165953	-3.534640	-0.373644
C	-2.272914	-2.814671	0.804071
C	-1.166970	-3.531964	0.375143
C	-1.179192	-5.000244	0.361237
C	1.183049	-5.004819	-0.361277
N	-0.000852	-5.649643	0.000652

O	2.182484	-5.639887	-0.666576
O	-2.171112	-5.648848	0.664223
C	-0.035958	-7.111071	0.009284
H	0.947456	-7.472611	-0.283107
H	-0.291331	-7.467333	1.009409
H	-0.794887	-7.464906	-0.691623
C	-3.537555	-0.870828	1.366773
C	-4.421928	-1.912887	1.685824
C	-3.897216	0.439056	1.715685
C	-5.130077	0.686665	2.297377
C	-5.668017	-1.671588	2.263699
C	-6.022118	-0.361650	2.554385
H	-6.340173	-2.492785	2.494724
H	-6.988202	-0.153399	3.004385
H	-3.215916	1.263702	1.544210
H	-5.401817	1.705503	2.555673
C	-3.534632	0.870849	-1.368151
C	-4.419944	1.911876	-1.687074
C	-3.892079	-0.439220	-1.718673
C	-5.665304	1.669504	-2.266103
C	-5.124169	-0.687975	-2.301499
H	-3.209742	-1.263081	-1.547593
C	-6.017492	0.359350	-2.558129
H	-6.338357	2.490019	-2.496951
H	-6.982996	0.150168	-3.008942
H	-5.394314	-1.706944	-2.560957
C	3.537555	0.870828	1.366773
C	4.421928	1.912887	1.685824
C	5.668017	1.671588	2.263699
C	3.897216	-0.439056	1.715685
C	5.130077	-0.686665	2.297377
H	3.215916	-1.263702	1.544210
C	6.022118	0.361650	2.554385
H	6.340173	2.492785	2.494724
H	6.988202	0.153399	3.004385
H	5.401817	-1.705503	2.555673
C	3.534632	-0.870849	-1.368151
C	4.419944	-1.911876	-1.687074

C	5.665304	-1.669504	-2.266103
C	3.892079	0.439220	-1.718673
C	6.017492	-0.359350	-2.558129
H	6.338357	-2.490019	-2.496951
C	5.124169	0.687975	-2.301499
H	3.209742	1.263081	-1.547593
H	6.982996	-0.150168	-3.008942
H	5.394314	1.706944	-2.560957
S	-3.775011	-3.514030	1.350764
S	3.775011	-3.513340	-1.350361
S	3.775011	3.514030	1.350764
S	-3.775011	3.513340	-1.350361

3b-*meso*:

N	5.626106	0.600056	0.000000
C	5.000180	0.420412	1.232647
C	5.000180	0.420412	-1.232647
C	3.554297	0.163169	1.217850
C	3.554297	0.163169	-1.217850
C	2.841515	0.136529	0.000000
C	2.882294	-0.196731	2.373069
C	2.882294	-0.196731	-2.373069
C	1.426235	0.050810	0.000000
C	0.729643	-0.080070	1.242160
C	1.477391	-0.460811	-2.365903
C	0.729643	-0.080070	-1.242160
C	1.477391	-0.460811	2.365903
C	-0.729634	0.080077	-1.242160
C	-1.426224	-0.050808	-0.000000
C	-0.729634	0.080077	1.242160
O	5.636759	0.483998	-2.274989
C	7.061392	0.875802	0.000000
O	5.636759	0.483998	2.274989
H	7.305432	1.441028	0.898020
H	7.627695	-0.060135	0.000000
H	7.305432	1.441028	-0.898020
C	-1.477385	0.460813	-2.365904
C	-1.477385	0.460813	2.365904

C	-2.841504	-0.136539	-0.000000
C	-2.882285	0.196718	-2.373069
C	-3.554286	-0.163187	-1.217850
C	-2.882285	0.196718	2.373069
C	-3.554286	-0.163187	1.217850
C	-5.000165	-0.420443	1.232648
C	-5.000165	-0.420443	-1.232648
N	-5.626097	-0.600067	-0.000000
O	-5.636747	-0.484005	-2.274989
O	-5.636747	-0.484005	2.274989
C	-7.061387	-0.875794	-0.000000
H	-7.305434	-1.441017	-0.898020
H	-7.305434	-1.441017	0.898020
H	-7.627677	0.060151	-0.000000
C	-1.070958	1.107121	3.615052
C	-2.113987	1.110719	4.554029
C	0.118165	1.776247	3.939370
C	0.270973	2.352544	5.189637
C	-1.962846	1.672977	5.821229
C	-0.756769	2.281291	6.138370
H	-2.779018	1.652196	6.537388
H	-0.620233	2.727231	7.119054
H	0.922753	1.850548	3.216993
H	1.198792	2.861417	5.431969
C	1.070954	-1.107109	3.615053
C	2.113984	-1.110721	4.554029
C	-0.118181	-1.776212	3.939373
C	1.962834	-1.672975	5.821231
C	-0.270998	-2.352503	5.189641
H	-0.922771	-1.850498	3.216997
C	0.756746	-2.281268	6.138373
H	2.779005	-1.652205	6.537390
H	0.620203	-2.727204	7.119058
H	-1.198826	-2.861359	5.431975
C	1.070954	-1.107109	-3.615053
C	2.113984	-1.110721	-4.554029
C	1.962834	-1.672975	-5.821231
C	-0.118181	-1.776212	-3.939373

C	-0.270998	-2.352503	-5.189641
H	-0.922771	-1.850498	-3.216997
C	0.756746	-2.281268	-6.138373
H	2.779005	-1.652205	-6.537390
H	0.620203	-2.727204	-7.119058
H	-1.198826	-2.861359	-5.431975
C	-1.070958	1.107121	-3.615052
C	-2.113987	1.110719	-4.554029
C	-1.962846	1.672977	-5.821229
C	0.118165	1.776247	-3.939370
C	-0.756769	2.281291	-6.138370
H	-2.779018	1.652196	-6.537388
C	0.270973	2.352544	-5.189637
H	0.922753	1.850548	-3.216993
H	-0.620233	2.727231	-7.119054
H	1.198792	2.861417	-5.431969
S	-3.615032	0.428850	3.940206
S	3.615037	-0.428875	3.940205
S	3.615037	-0.428875	-3.940205
S	-3.615032	0.428850	-3.940206

3b-*P*-TS:

C	-2.464585	2.852001	-0.402798
C	-2.521632	1.600041	0.292160
C	-1.356201	0.792724	0.340158
C	-0.096112	1.490363	0.142831
C	1.173374	0.828793	0.191246
C	2.346315	1.517362	-0.166064
C	2.317366	2.942182	-0.182294
C	-2.161105	-2.678208	-0.977075
C	-2.358887	-1.635751	-0.012702
C	-1.306104	-0.704690	0.185179
C	-0.005937	-1.288371	-0.001780
C	1.167593	-0.609995	0.424203
C	2.229719	-1.380954	0.904032
C	2.338465	-2.736951	0.469809
C	4.283529	-2.047882	1.867349
C	3.352180	-1.004627	1.753892

C	3.569559	0.178703	2.471955
C	5.438383	-1.917565	2.637851
C	4.714502	0.314691	3.239958
C	5.651374	-0.724606	3.314834
H	6.154837	-2.730755	2.707467
H	6.548200	-0.601153	3.914589
H	4.887091	1.237842	3.784563
H	2.851290	0.990514	2.422282
C	3.680998	1.045467	-0.565182
C	4.614028	2.095788	-0.570981
C	5.954817	1.896626	-0.898117
H	6.658100	2.723885	-0.872321
C	4.102257	-0.199190	-1.056854
H	3.396876	-1.010096	-1.182632
C	6.362237	0.631607	-1.296214
C	5.426268	-0.402460	-1.410034
H	5.732862	-1.376063	-1.779951
H	7.401842	0.456059	-1.556160
C	-4.481688	-2.721831	-0.113927
C	-3.628463	-1.918388	0.663594
C	-3.867366	-1.869770	2.040701
C	-5.699820	-3.182873	0.379886
C	-5.991514	-2.975499	1.724009
C	-5.042686	-2.386802	2.565326
H	-6.362114	-3.774849	-0.244636
H	-6.920109	-3.358646	2.136676
H	-5.217313	-2.347177	3.636375
H	-3.102178	-1.478322	2.701724
C	-4.812356	2.263074	0.116766
C	-3.892849	1.436308	0.788126
C	-4.307112	0.877729	1.996667
H	-3.568848	0.448087	2.657143
C	-6.161525	2.296546	0.458986
H	-6.856549	2.920565	-0.094836
C	-5.637800	0.938551	2.383921
C	-6.577260	1.591849	1.582627
H	-5.945468	0.484227	3.320301
H	-7.621513	1.618844	1.879532

C	-0.902254	-3.183040	-1.231428
C	0.161945	-2.595054	-0.520483
C	1.375614	-3.291408	-0.360784
C	-1.275448	3.519612	-0.588637
C	-0.088390	2.875196	-0.191626
C	1.113464	3.615044	-0.230356
C	1.096117	5.064910	-0.473345
O	2.087514	5.764498	-0.323730
C	-1.261820	4.888233	-1.130311
O	-2.243969	5.382656	-1.662939
C	1.557395	-4.598331	-1.001344
O	2.562526	-5.275037	-0.834284
C	-0.686548	-4.396818	-2.029478
O	-1.539499	-4.834970	-2.785588
N	-0.087931	5.615323	-0.950394
N	0.543562	-5.038490	-1.855139
C	-0.057547	7.028465	-1.320412
C	0.787397	-6.294765	-2.561285
H	-1.045952	7.299593	-1.685642
H	0.837887	-7.119378	-1.846213
H	-0.031537	-6.451984	-3.260198
H	1.739154	-6.234277	-3.091838
H	0.208591	7.628379	-0.447812
H	0.692126	7.191148	-2.097971
S	3.830950	-3.489914	0.963421
S	-3.674550	-3.371372	-1.542046
S	-4.028565	3.445050	-0.918274
S	3.897753	3.674507	-0.275897

3b-*M*-TS:

C	-2.289777	2.960754	-0.199069
C	-2.326132	1.536133	-0.174095
C	-1.162957	0.841677	0.197089
C	0.112493	1.492699	0.156206
C	1.364611	0.780560	0.351255
C	2.540177	1.574681	0.303463
C	2.492371	2.830426	-0.385321
C	-2.378852	-2.703588	0.474520

C	-2.249524	-1.351500	0.914426
C	-1.174594	-0.596192	0.435846
C	-0.012252	-1.287819	0.005635
C	1.296421	-0.716656	0.189932
C	2.338110	-1.654249	-0.015926
C	2.126822	-2.694098	-0.979039
C	4.456021	-2.746683	-0.145821
C	3.612859	-1.950342	0.648697
C	3.859174	-1.916018	2.023166
C	5.676977	-3.213074	0.333566
C	5.037564	-2.442182	2.534625
C	5.978350	-3.020808	1.679183
H	6.333835	-3.800359	-0.301005
H	6.909789	-3.409752	2.079861
H	5.220120	-2.415297	3.604679
H	3.098298	-1.529221	2.692399
C	3.913688	1.395663	0.790153
C	4.836679	2.219833	0.119361
C	6.187824	2.242882	0.454629
H	6.883217	2.866188	-0.099505
C	4.332245	0.826586	1.993023
H	3.596788	0.393823	2.654867
C	6.604997	1.529592	1.571731
C	5.665394	0.876907	2.373344
H	5.975783	0.412896	3.304016
H	7.651115	1.547636	1.862713
C	-4.323516	-1.984050	1.856925
C	-3.369506	-0.959831	1.760722
C	-3.569295	0.220427	2.489844
C	-5.483501	-1.836936	2.617281
C	-5.678327	-0.646849	3.303563
C	-4.718009	0.372588	3.248299
H	-6.216502	-2.636410	2.672733
H	-6.577892	-0.510406	3.896334
H	-4.875047	1.292833	3.802450
H	-2.833876	1.017445	2.457176
C	-4.587569	2.120665	-0.600435
C	-3.660098	1.066589	-0.580668

C	-4.083550	-0.179556	-1.065877
H	-3.381179	-0.994304	-1.182719
C	-5.926966	1.925820	-0.934853
H	-6.626195	2.756770	-0.919922
C	-5.406806	-0.379868	-1.424748
C	-6.338408	0.659258	-1.324575
H	-5.715491	-1.354900	-1.789091
H	-7.377355	0.486480	-1.588908
C	-1.415053	-3.278606	-0.342727
C	-0.194102	-2.595626	-0.505010
C	0.864326	-3.198081	-1.214016
C	-1.081338	3.627899	-0.231593
C	0.115681	2.878453	-0.176330
C	1.310880	3.511218	-0.566552
C	1.308281	4.882208	-1.101975
O	2.282305	5.373088	-1.651711
C	-1.056607	5.076326	-0.485461
O	-2.061030	5.767772	-0.395679
C	0.639416	-4.421057	-1.993918
O	1.481733	-4.868751	-2.756828
C	-1.606878	-4.593433	-0.966585
O	-2.624939	-5.251061	-0.801832
N	-0.582722	-5.066619	-1.790139
N	0.155390	5.629711	-0.886293
C	-0.827664	-6.329215	-2.484654
C	0.172523	7.040747	-1.265118
H	-1.278718	-7.034994	-1.786274
H	0.064440	7.159530	-2.348795
H	-0.659518	7.532570	-0.767270
H	1.128340	7.466058	-0.962055
H	-1.513848	-6.170940	-3.320306
H	0.122777	-6.706345	-2.856619
S	3.631847	-3.384603	-1.569822
S	4.058149	3.411758	-0.907393
S	-3.866388	3.698290	-0.312740
S	-3.892216	-3.428255	0.946578

Supplementary References

1. Wu, J.; He, D.; Zhang, L.; Liu, Y.; Mo, X.; Lin, J.; Zhang, H.-J., Direct Synthesis of Large-Scale Ortho-Iodinated Perylene Diimides: Key Precursors for Functional Dyes. *Org. Lett.* **2017,** *19* (19), 5438-5441.
2. Chai, J. D.; Head-Gordon, M., Long-range corrected hybrid density functionals with damped atom-atom dispersion corrections. *Phys. Chem. Chem. Phys.* **2008,** *10* (44), 6615-6620.
